# Robust and replicable measurement for prepulse inhibition of the acoustic startle response

**DOI:** 10.1038/s41380-020-0703-y

**Published:** 2020-03-06

**Authors:** Eric A. Miller, David B. Kastner, Michael N. Grzybowski, Melinda R. Dwinell, Aron M. Geurts, Loren M. Frank

**Affiliations:** 1 Kavli Institute for Fundamental Neuroscience and Department of Physiology, University of California, San Francisco, CA 94158 USA; 2 Department of Psychiatry, University of California, San Francisco, CA 94143 USA; 3 Department of Physiology, Medical College of Wisconsin, Milwaukee, WI USA; 4 Howard Hughes Medical Institute, San Francisco, CA USA

**Keywords:** Neuroscience, Biological techniques

## Abstract

Measuring animal behavior in the context of experimental manipulation is critical for modeling, and understanding neuropsychiatric disease. Prepulse inhibition of the acoustic startle response (PPI) is a behavioral phenomenon studied extensively for this purpose, but the results of PPI studies are often inconsistent. As a result, the utility of this phenomenon remains uncertain. Here, we deconstruct the phenomenon of PPI and confirm several limitations of the methodology traditionally utilized to describe PPI, including that the underlying startle response has a non-Gaussian distribution, and that the traditional PPI metric changes with different stimuli. We then develop a novel model that reveals PPI to be a combination of the previously appreciated scaling of the startle response, as well as a scaling of sound processing. Using our model, we find no evidence for differences in PPI in a rat model of Fragile-X Syndrome (FXS) compared with wild-type controls. These results in the rat provide a reliable methodology that could be used to clarify inconsistent PPI results in mice and humans. In contrast, we find robust differences between wild-type male and female rats. Our model allows us to understand the nature of these differences, and we find that both the startle-scaling and sound-scaling components of PPI are a function of the baseline startle response. Males and females differ specifically in the startle-scaling, but not the sound-scaling, component of PPI. These findings establish a robust experimental and analytical approach that has the potential to provide a consistent biomarker of brain function.

## Introduction

Prepulse inhibition of the acoustic startle response (PPI) is a reduction in the magnitude of the acoustic startle response when a weak, non-startling sound—the prepulse—precedes an intense, potentially startling, sound [1–3]. Changes in PPI have been linked to various neuropsychiatric disorders, such as schizophrenia [4–9], obsessive compulsive disorder [10–13], Tourette’s syndrome [14, 15], autism-spectrum disorder [16–18], and post-traumatic stress disorder [19, 20]. As such, PPI has been promoted as a potential biomarker of brain function in the context of disease [21, 22]. Furthermore, since PPI can be studied in both humans and laboratory animals, it offers a translational methodology for generating mechanistic insights into those diseases [23–25].

However, published PPI results are often inconsistent with one another [26], potentially undermining the utility of the phenomenon. The source of these inconsistencies has been associated with differences between experimental conditions [27], analytical methods [25], or factors such as strain [28, 29], age [30–32], sex [13, 33–35], reproductive cycle [36, 37], species [38–40], acute disease state [41], habituation [42], socialization [43, 44], and the baseline startle response [45]. Consequently, there is a pressing need for an approach that could consistently identify real differences among groups. We therefore sought to deconstruct the phenomenon of PPI to develop a more accurate methodology for capturing the way in which a prepulse stimulus modifies the acoustic startle response.

The phenomenon of PPI is distinct from the specific methodology used for measuring it. The traditional PPI methodology makes four assumptions: (1) the startle response can be accurately measured with a small number of trials per animal; (2) the startle response has an approximately Gaussian distribution, allowing the use of the mean startle response as the basis of the PPI metric, and for comparison of PPI between groups using statistical tests such as ANOVA that assume a Gaussian noise model; (3) PPI is consistent across startle sound levels, enabling the measurement of PPI at a single startle level instead of necessitating a full measurement of the startle function; (4) PPI is independent of the baseline startle response, allowing for a direct combination of PPI results between animals.

Using data from 72 rats across more than 100 stimuli, for a total of over 300,000 trials, we replicated previous work demonstrating that the aforementioned assumptions do not hold. Specifically, our findings confirm that (1) the startle response within animals is highly variable [46]; (2) the startle response within animals has a non-Gaussian distribution that is better represented by a log-normal distribution [47]; (3) the traditional metric used for PPI systematically decreases as a function of sound level [48]; (4) PPI is also a function of the baseline startle response [45].

These problems were previously identified in individual studies, but a systematic approach to address them is lacking. Therefore, we developed a novel analytical model of PPI characterized by a scaling of both the startle response and the startle sound. Using cross-validation, we found that our model better fits the data than the implicit model underlying the traditional PPI metric. Thus, the phenomenon of PPI consists of both a reduction of the startle response (startle scaling) and a reduction of sound processing (sound scaling). Our model also addresses all of the aforementioned limitations of the traditional PPI methodology.

Using our model, and data from multiple cohorts of animals, we conclude that *Fmr1*-knockout (KO) rats—rats missing the gene silenced in Fragile-X Syndrome (FXS)—do not differ from wild-type (WT) rats in PPI. In contrast, we found that WT female rats differ from WT male rats in the startle-scaling, but not the sound-scaling, component of PPI. These experimental findings, grounded in a formal mathematical model, demonstrate the utility of our approach to yield robust and replicable findings about the relationship between PPI and genetic or experimental manipulations. As such, this approach could be used to clarify the inconsistent PPI results in the context of brain diseases, such as those reported in mouse models of FXS [39, 49–56].

## Materials and methods

### Animals

All experiments were conducted in accordance with the Medical College of Wisconsin and University of California San Francisco Institutional Animal Care and Use Committee, and US National Institutes of Health guidelines. Rat datasets were collected from Long Evans rats that were fed standard rat chow (LabDiet 5001).

The *Fmr1* KO rats were males with a CRISPR/SpCas9 knockout of *Fmr1* on a Long Evans background generated at the Medical College of Wisconsin. Briefly, a CRISPR targeting the *Fmr1* exon 8 sequence 5′-GGTCTAGCTATTGGTACTCA**TGG**-3′ (PAM in bold) was injected into Crl:LE embryos (Charles River Laboratories). Two mutant strains were generated (LE-*Fmr1*^*em2Mcwi*^ and LE*-Fmr1*^*em4Mcwi*^) (RGDIDs: 11553873 and 11553875) with mutations in *Fmr1*. LE-*Fmr1*^*em2Mcwi*^ harbors a net 2-bp insertion, while LE*-Fmr1*^*em4Mcwi*^ harbors a 2-bp deletion mutation at the SpCas9 cleavage site (Fig. S1a). Both mutations are predicted to cause frameshifts, and complete loss of FMR1 expression was confirmed by Western blot (Fig. S1b). Knockout rats were a similar size to their wild-type (WT) counterparts, but like *Fmr1*-knockout mice [57, 58], knockout rats had increased testicular weights at 30 days of age (*p* < 0.04) (Fig. S1c). Breeding colonies for both strains are maintained at the Medical College of Wisconsin in continuous backcross of heterozygous females to vendor Crl:LE males at each generation to avoid inbreeding and genetic drift.

We used a total of 72 rats in five different cohorts (Supplementary Table 1). The first *Fmr1* cohort consisted of ten KO males and eight WT littermate males from the LE-*Fmr1*^*em2Mcwi*^ strain. This cohort was from four different litters. They underwent two rounds of PPI experimentation, separated by 3–4 months, and they were aged 11–12 months at the time of the first PPI experimentation and 14–15 months at the time of the second PPI experimentation. Between the two experiments, one of the *Fmr1* KO male rats developed a tumor and was euthanized.

The second *Fmr1* cohort consisted of nine KO males and nine WT littermate males from the LE*-Fmr1*^*em4Mcwi*^ strain. This cohort was from four different litters. They underwent one round of PPI experimentation, and they were aged 4–5 months at the time of the PPI experimentation.

The third cohort consisted entirely of WT males consisting of 12 rats. This cohort was from 2 litters. They underwent two rounds of PPI experimentation, and were 9–11 months at the time of the first experimentation and 13–15 months at the time of the second experimentation. As these rats had similar behavioral experiences as the first cohort of *Fmr1* rats, and were experimented on at roughly the same time, we included them in our analysis of the effects of *Fmr1* KO on PPI. Our conclusions remain unchanged whether or not we included these animals.

The fourth cohort was one of two WT male–female cohorts, and consisted of six males and six females. This cohort was from 2 litters. They underwent two rounds of PPI experimentation separated by 2 months. They were aged 4–5 months at the time of the first PPI experimentation, and 6–7 months at the time of the second PPI experimentation.

The fifth cohort was the second WT male–female cohort, and consisted of six males and six females. This cohort was from 2 litters. They underwent two rounds of PPI experimentation separated by 2 months. They were aged 3–4 months at the time of the first PPI experimentation, and 5–6 months at the time of the second PPI experimentation.

During the collection of the data, experimenters were blind to genotype (*Fmr1* KO vs. WT), but not blind to sex, as that would not be possible. There was no randomization as the genotype and sex defined the groupings of the animals.

### Data collection and analysis

Experiments were conducted using four SR-Lab startle systems (San Diego Instruments). The systems were calibrated with a digital sound meter in the center of the test chamber. Each experiment consisted of 12 sessions, with the exception of two experiments that had 6 sessions. On the day prior to the first session, each rat was placed in the apparatus for 1 h of constant background sound for initial habituation to the apparatus. Each session began with 5 min of background sound, followed by five habituation trials of a sound 50 dB above the background sound and no prepulse sound. After the habituation trials, sessions consisted of either five or seven repeats of 21–48 different stimuli, randomly ordered and separated by intertrial intervals randomly drawn from the range of 5–15 s. Rats completed 2–3 sessions per day, and in total, each rat received either 60 or 84 (12-session experiments) or 28–32 (6-session experiments) repeats of each stimulus.

A stimulus was defined by three parameters: the startle sound level, the prepulse sound level, and the delay time between the prepulse and startle sounds. We used a range of startle sounds that elicited startle responses covering the animals’ full startle response functions in order to accurately fit the model to the data (see Supplementary Methods). The startle sound level varied between 0 and 60 dB above background; the prepulse sound level varied between 0 and 18 dB above background; the delay time varied between 50 and 200 ms. Prepulse and startle sounds were white noise bursts lasting for 20 and 40 ms, respectively. The delay time was calculated from the time of prepulse onset. The background sound was either 70 or 77 dB, depending on the experiment. We confirmed that all of our results are the same between the 70- and 77-dB background sounds, and the animals did not significantly startle to the prepulse prior to the startle sound onset (see Supplementary Methods).

The raw accelerometer readings were first normalized to account for different gains of the startle systems. For each session and rat, we fit a Gaussian distribution to the distribution of accelerometer readings for the first 100 ms of every trial. This is always before the presentation of the startle stimulus, and therefore represents a baseline (Fig. S2a, b). Each accelerometer reading was then *z*-score normalized by subtracting the mean and dividing by the standard deviation of the Gaussian fit.

Following this normalization, we identified the maximal value within 100 ms following the startle sound for each trial (Fig. S2c). We then averaged these maximal values across trials at a given stimulus, which we define as the movement of the animal to that stimulus. The movement was then used to compute the standard metric for PPI

1 $${\mathrm{PPI}}_{{\mathrm{ratio}}} \,=\, \frac{{m_b - m_p}}{{m_b}} \,=\, 1 - \frac{{m_p}}{{m_b}}$$

where *m*_*b*_ is the movement of the animal in response to the startle sound alone, i.e., the baseline startle response, and *m*_*p*_ is the movement to the startle sound preceded by a prepulse sound.

We combined the trial repeats of a given stimulus across all of the sessions of an experiment, as we observed only minor changes in baseline startle response between the first and second halves of experiments, such that the mean changes in startle were smaller than the interquartile range between animals, and we did not observe between-trial dependencies (see Supplementary Methods). Furthermore, PPI is not thought to habituate across trials [7, 25], although PPI has been found to increase with repeated testing [59]. Correspondingly, we found some indication for changes in PPI within animals across all of the repeats. However, the changes were small compared with the differences between animals (see Supplementary Methods), so we therefore combined all of the trial repeats for the development of our methodology.

### Functional model of PPI

We describe an animal’s baseline startle responses with the equation *m*(*s*) = *m*_0_ + *N*(*s*), where *m* is the movement as a function of a startle sound, *N*(*s*) is a monotonically increasing function of *s*, the startle sound level, and *m*_0_ is the baseline movement independent of sound. We define this equation as the animal’s baseline startle curve, corresponding to movement in the absence of a prepulse sound.

We then introduce scaling parameters to describe how the baseline startle curve is modified by different prepulse conditions. Here, a prepulse condition is defined by the intensity of the prepulse sound, and the delay between the prepulse and startle sounds. Note that each prepulse condition was paired with many different startle sounds, *s*. We never combine data from experiments that varied the prepulse sound with those that varied the delay time, always treating them as separate conditions.

First, we introduce a parameter, *α*_*c*_, corresponding to the scaling of the startle response due to a prepulse condition, *c*. A model with just startle scaling is the model that implicitly underlies the traditional PPI_ratio_ metric. After subtracting the baseline movement, *m*_0_, we are left with the following model with just startle scaling:

2 $$m_c\left( s \right) = \alpha _cN\left( s \right).$$

Second, we introduced a parameter, *β*_*c*_, corresponding to the scaling of the startle sound in a specific prepulse condition. This gives us the following model with both startle- and sound scaling:

3 $$m_c\left( s \right) = \alpha _cN\left( {\beta _cs} \right).$$

Finally, we used a sigmoid function as the monotonically increasing function, *N*(·), at the basis of our model. This sigmoid describes the specific functional form of the baseline startle curve, i.e., the startle responses without a prepulse sound:

4 $$N\left( s \right) = \frac{{m_{{\mathrm{max}}}}}{{1 + e^{ - r\left( {s - s_0} \right)}}},$$

where *s* is the startle sound level, *m*_max_ is the maximal movement due to a startling sound, i.e., the saturation point, *s*_0_ is the sound at which the animal startles at 50% of maximal, and *r* reflects the slope of the sigmoid, describing how rapidly the startle response changes from zero to maximal.

Thus, in total, this model contains 3 + 2n parameters per animal, where *n* is the number of prepulse conditions to which the animal was exposed. There are three parameters for the baseline startle curve (*m*_max_,*r*, *s*_0_), and two scaling parameters for each prepulse condition (*α*_*c*_, *β*_*c*_). The baseline startle curve (Eq. 4) is modified by different prepulse conditions, c, according to the scaling parameters *α*_*c*_ and *β*_*c*_ (Eq. 3), which range between 0 (100% scaling) and 1 (0% scaling). This is a formal model of PPI that can be fit to data from individual animals. We separately tried fitting the model without bounds on *α*_*c*_ and *β*_*c*_, but this did not improve the cross-validated accuracy (see Supplementary Methods). Therefore, we chose to use the bounded fits, as that prevented compensation between the parameters, allowing for more interpretable results.

We fit all of the data for a given animal with a single fitting routine, minimizing the total root mean-squared error (RMSE) between the model and the data across all stimuli. Initial conditions for the scaling parameters were no scaling (i.e., *α*_*c*_ = *β*_*c*_ = 1 for all *c*); initial conditions for the baseline startle curve were set to the parameters that best fit the baseline data alone, which we obtained by separately fitting a sigmoid to the baseline data. For ease of comparison with the traditional PPI_ratio_ metric, the scaling parameters were converted to percentage scaling using the equations 100 × (1 − *α*_*c*_) and 100 × (1 − *β*_*c*_).

We determined whether both startle scaling and sound scaling made significant contributions to the fit of the model using cross-validation. Specifically, we cross-validated the model with both startle- and sound scaling (Eq. 3), and compared against the model with only startle scaling (Eq. 2) by training each model on 80% of the data, and then testing on the remaining 20% of holdout data. For each rat in each experiment, we conducted 100 iterations of cross-validation on randomly selected training data and the remaining testing data. In each iteration, we computed a normalized RMSE between the models and the testing data, such that the difference between the model and the data at each stimulus was normalized by the standard error at that stimulus. Then, for each rat in each experiment, we computed the average normalized cross-validation error across all 100 iterations for both models.

We sought to evaluate whether the parameters obtained from an individual animal’s model fit were distinct from the parameters that best fit the other animals. To do that we swapped the set of parameters that best fit each animal’s startle curves in the same experiment, and computed the new fitting errors for each rat’s data (Fig. S3a). For example if A is the parameter set that best fit rat 1 and B is the parameter set that best fit rat 2, we computed the error from fitting rat 1’s startle data with rat 2’s optimal parameter set, B, and compared that to the minimal error that we get from using A. We repeated that swap for all animals that were part of the same experiment.

To evaluate the estimation precision of each parameter, we computed 90% confidence intervals for each parameter by fitting the model 10,000 times to jittered data, in which each startle data point was jittered by a random draw from a Gaussian with standard deviation equal to the standard error of the startle (Fig. S3b). The 90% confidence intervals were defined as spanning the 5–95th percentile of the parameter values across all of the fits to jittered data for a given rat.

### Group differences in model parameters

The model fits produce 3 + 2n parameters per animal, where *n* is the number of prepulse conditions to which the animal was exposed. We separately analyzed the experiments that primarily varied the prepulse sound level from the experiments that primarily varied the delay time between the prepulse and startle sounds, as these are distinct manipulations. We determined whether groups of animals differed using a linear discriminant analysis (LDA), which finds the hyperplane that best linearly separates the two groups in the high-dimensional space defined by all of the parameters that are being compared. For each prepulse condition, we performed the LDA using the three baseline parameters and the two scaling parameters that defined the prepulse condition.

With LDA, two groups are linearly separable if there exists a hyperplane, such that the data from the two groups consistently fall on opposite sides of the hyperplane. To visualize this, we projected the data onto the vector orthogonal to the hyperplane, called the linear discriminate (LD), since by definition this is the vector that best linearly separates the groups. To evaluate the significance of the LDA, we computed a permutation test on the mean absolute distance from the LDA hyperplane with 10,000 iterations of randomly permuted group labels. In addition, leave-one-out cross-validation was computed for each prepulse condition using a permutation test with 10,000 iterations. We define group differences in the model parameters as significantly great mean absolute distance from the LDA hyperplane (*p* < 0.05, permutation test) and cross-validated classification accuracy (*p* < 0.05, permutation test) in a significant number of prepulse conditions after controlling for multiple comparisons (*p* < 0.05, bootstrapped ratio test).

### PPI versus baseline startle correlations

We define the baseline threshold as the minimum sound required for an animal to startle at 5% of its startle saturation (*m*_max_). For each prepulse condition, we computed Pearson’s *r* and *r*^2^ values across animals for startle scaling versus baseline saturation, and for sound scaling versus baseline threshold. We sought to ensure that the correlations were robust to potential variability in parameter estimates. Therefore, we evaluated whether the correlations that we observed could have been due to noise in the startle data that lead to imprecision in the parameters for the best fits of the model. We refit all of the startle curves 10,000 times after jittering each startle data point by a random sample from a Gaussian with a mean equal to the mean of the data and a standard deviation equal to the standard error of the mean. For each of these 10,000 fits to jittered data we recomputed the correlations between the parameters of the model for each prepulse condition (Fig. S3c). We then evaluated the robustness of the observed correlations by measuring the likelihood of seeing a correlation of *r* = 0.

A potential concern with sigmoid models is that parameters can compensate for each other, and thereby create correlations in the parameters that do not reflect correlations in the data, but rather reflect a space across which there are relatively similar fitting errors. To ensure that the correlations were not caused by correlated estimates of the model parameters, we compared our across-animal correlations with a distribution of within-animal correlations generated from re-fitting the model 1000 times to jittered data. Each data point was jittered by a Gaussian with standard deviation equal to the standard error of the data point. We then compared the distribution of within-animal correlations across all of the fits to jittered data with our observed across-animal correlations (Fig. S3d).

### Group differences in PPI

For groups of animals where the prepulse condition LDA analysis revealed a difference, we carried out a second set of analyses to understand the source of the differences while adjusting for correlations between PPI and the baseline startle. For the comparison between baseline startle parameters and sound- and startle scaling, we computed ANCOVAs, including a group-by-baseline interaction term. This interaction term was used to confirm homogeneity of slopes between groups. We also checked for group differences in the baseline parameters using *t* tests.

We did not include ANCOVAs for two prepulse conditions in which WT male and female rats differed in baseline threshold, as the ANCOVA is inappropriate in the presence of nonrandom group differences in the covariate [60]. However, we continued to use ANCOVAs for all other prepulse conditions, since, as a whole, the groups did not differ on either baseline covariate. Finally, group effects on startle- and sound scaling were analyzed using ANCOVAs without a group-by-baseline interaction term. We define group differences in the sound-scaling or startle-scaling components of PPI as a significant main effect of group (*p* < 0.05, ANCOVA) in a significant number of prepulse conditions after controlling for multiple comparisons (*p* < 0.05, bootstrapped ratio test).

## Results

We first set out to understand potential causes of inconsistencies in PPI results in the literature. Studies of the *Fmr1* KO mouse have reported increases [39, 49–51, 56], decreases [52, 53], or no difference [54, 55] in PPI compared with WT, and one study concluded that *Fmr1* KO mice show the opposite PPI result compared with humans with FXS [39]. As PPI had not been explored in *Fmr1* KO rats, we initially asked whether these inconsistencies could be due to species differences. At the same time, we noted that in the previous studies, only a small number (<10) of repeats of any given stimulus was used, raising the possibility that variability in PPI measurements also contributed.

**Fig. 1:**
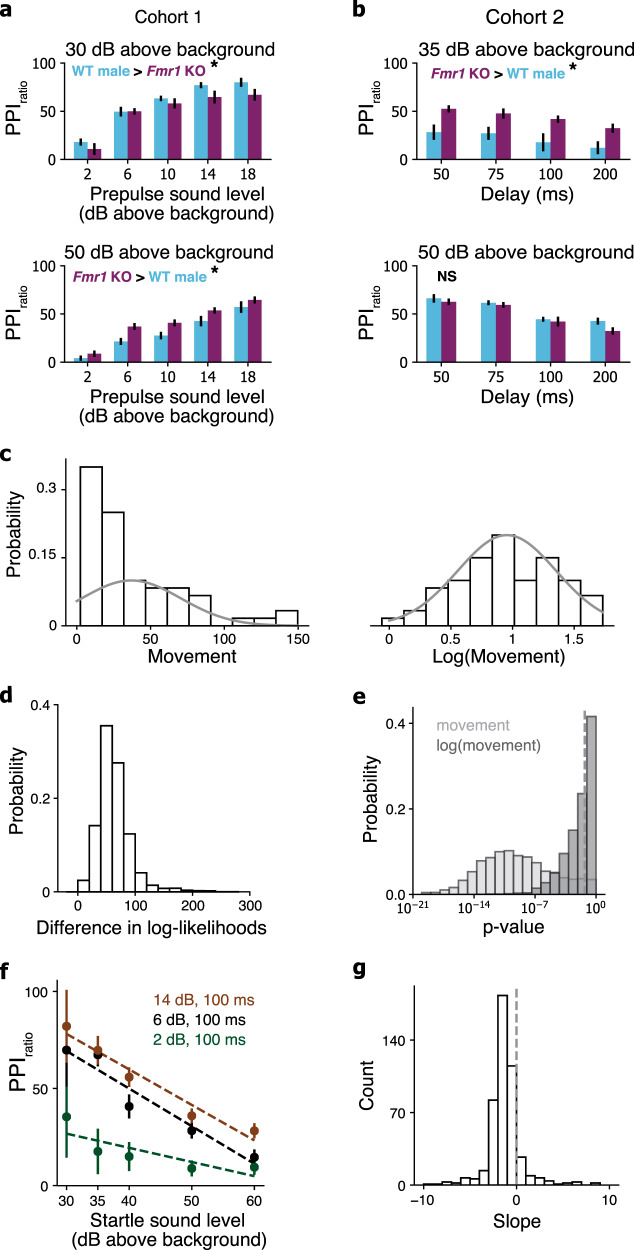
Inconsistencies in standard PPI_ratio_ measurement. **a** PPI_ratio_ from the first *Fmr1* cohort of male rats. Blue: WT (*n* = 7) and red: *Fmr1* KO (*n* = 9) across different prepulse sounds with a constant 100 ms delay and startle sounds of 30 dB above background (top) or 50 dB above background (bottom). **b** PPI_ratio_ from the second *Fmr1* cohort of male rats. Blue: WT (*n* = 9) and red: *Fmr1* KO (*n* = 9) across different delays with a constant prepulse of 14 dB above background and startle sounds of 35 dB above background (top) or 50 dB above background (bottom). For a and b, significant group differences, indicated by an asterisk, were defined as a two-way ANOVA with *p* < 0.05. See Supplementary Methods for further PPI_ratio_ analysis on these data. **c** Example probability distribution of gain-normalized movement (see Fig. S2) (left) and log_10_ of gain-normalized movement (right) for one rat to a startle sound of 40 dB above background with no prepulse. Solid curves are Gaussian functions with mean and standard deviation equal to those of the data and height equal to the height of the bin containing the mean. **d** Distribution of the differences in log-likelihood of the movement data under a log-normal distribution and the log-likelihood of the movement data under a Gaussian distribution across all rats and stimuli. Positive value indicates that the log-normal distribution had a larger log-likelihood. **e** Distribution of the Shapiro–Wilks normality test *p* values across all rats and stimuli for the data before log transformation (light gray) and after log transformation (dark gray). Dotted vertical line shows *p* = 0.05. Smaller *p* values indicate greater probability of rejecting the null hypothesis that the data is drawn from a Gaussian distribution. **f** PPI_ratio_ using log-transformed movement data for one rat (same rat as Fig. 1c) across five different startle sound levels (*x*-axis), three different prepulse sounds (colors), and a constant 100 ms delay. Bars show the standard error of the mean. Dotted lines show linear regressions for each prepulse sound level. **g** Distribution of linear regression slopes of PPI_ratio_ versus startle sound level (dotted lines in Fig. 1f) across all rats and stimuli. Dotted line shows slope of 0.

We therefore collected data from 28 to 84 (median 60) repeats of each stimulus in each individual rat (see Supplementary Table 1). Strikingly, even with the larger number of trials, we reproduced the inconsistent results found in mice, both within the same cohort of animals at different sound levels and across different cohorts of animals at similar sound levels. In the first cohort of rats, we varied the prepulse- and the startle sound, while keeping the delay between the prepulse and the startle sound constant. We found stimuli where the two groups differed: *Fmr1* KO rats had a lower PPI_ratio_ than WT rats when the startle sound was 30 dB above baseline (*p* < 0.04, two-way ANOVA) (Fig. 1a). In contrast, *Fmr1* KO rats had a greater PPI_ratio_ than WT rats when the startle sound was 50 dB above baseline (*p* < 10^−3^, two-way ANOVA) (Fig. 1a). In the second cohort of rats, we varied the delay between the prepulse and the startle sound, while keeping the prepulse sound constant. We found that *Fmr1* KO rats had a greater PPI_ratio_ than WT rats when the startle sound was 35 dB above baseline (*p* < 10^−4^, two-way ANOVA) (Fig. 1b). In contrast, there was no difference in PPI_ratio_ when the startle sound was 50 dB above baseline (*p* > 0.09, two-way ANOVA) (Fig. 1b), and the trend was in the opposite direction from the 35 dB stimulus. We found no group-by-prepulse condition interactions (*p* > 0.05, two-way ANOVA).

PPI_ratio_ was also inconsistent between cohorts at similar sound levels. In cohort 1 at 30 dB above baseline, *Fmr1* KO rats had a lower PPI_ratio_ than WT animals (*p* < 0.04, two-way ANOVA), but in cohort 2 at 35 dB above baseline, *Fmr1* KO rats had a greater PPI_ratio_ than WT animals (*p* < 10^−4^, two-way ANOVA). In cohort 1 at 50 dB above baseline, *Fmr1* KO rats had a greater PPI_ratio_ than WT animals (*p* < 10^−3^, two-way ANOVA), but in cohort 2 at 50 dB above baseline, there was no difference between *Fmr1* KO and WT animals (*p* > 0.09, two-way ANOVA), and the trend was in the opposite direction from cohort 1. Thus, we found inconsistent PPI_ratio_ results within and between cohorts, showing that *Fmr1* KO rats exhibit similarly mixed PPI_ratio_ results as seen in *Fmr1* KO mice.

Interestingly, animals from cohort 1 had significantly lower baseline startle thresholds than animals from cohort 2 (*p* < 10^−7^, *t* test). Although the absolute sounds are similar, the stimuli represent different parts of the startle curve for animals in the two cohorts. This highlights a problem with PPI_ratio_, as it could be comparing very different parts of the underlying startle curves.

### Invalid assumptions underlie the PPI_ratio_ metric

Previous work identified two additional factors that could contribute to inconsistencies in PPI_ratio_ results: an incorrect assumption of an underlying Gaussian distribution [47], and an incorrect assumption about the consistency of PPI_ratio_ across different startle sounds [48]. Whether these issues are specific to the datasets examined in that past work or more general has not been established. We therefore asked if we could replicate these findings in our cohorts.

Both findings replicated. First, we found that the data are not consistent with an underlying Gaussian distribution but were instead more consistent with a log-normal distribution. (Fig. 1c, d). Data from only 4.51% of all of the stimuli across all animals were consistent with a Gaussian distribution (Fig. 1e) (*p* > 0.05, Shapiro–Wilks test). In contrast, 48.1% of the stimuli across all of the animals were consistent with a normal distribution after taking the log of the values, i.e., consistent with a log-normal distribution (Fig. 1e) (*p* > 0.05, Shapiro–Wilks test). This deviation from Gaussian is a problem for two reasons: (1) PPI_ratio_ uses a mean within animals as the primary measure of central tendency and (2) statistical tests commonly used for comparing PPI between groups, such as ANOVA, assume Gaussian distributions of parameters. While the log-normal is not a perfect fit, it was a better fit than a Gaussian distribution across all stimuli and rats (Fig. 1d), and it represents a good balance between fit and interpretability. We therefore chose to take the log of the max startle as the basis for our PPI measurements [47].

Second, we also confirmed that the traditional PPI_ratio_ measure is not the same across different startle sounds, given a constant prepulse condition [48]. If the PPI_ratio_ extracts a core feature of the phenomenon of PPI, then the ratio should be consistent across changes in the denominator, here the startle without a prepulse (Eq. 1). However, we found that not to be the case. Even when using the more accurate log-normal representation of the data, PPI_ratio_ systematically decreases as a function of increasing sound level (Fig. 1f). This decrease was seen in 422/488 (86.5%) of prepulse conditions across the 72 rats (Fig. 1g). Thus, understanding the phenomenon of PPI requires measuring it across different startle sounds.

### A new analytical model for PPI

The phenomenon of PPI is distinct from the specific metric used to measure it. The phenomenon of PPI is the change to the startle response due to the presence of a prepulse. We can think of a high-dimensional surface that describes the way in which an animal startles under all stimuli [61]. The axes of the surface are all of the factors that can change the startle response, such as the loudness of the startle sound, the loudness of the prepulse sound, and the delay between the prepulse and startle sounds. A full description of the phenomenon of PPI would be a functional description of that entire surface.

PPI_ratio_ attempts to capture the phenomenon by comparing two points in that surface: the magnitude of the startle without a prepulse and the magnitude of the startle in the presence of the prepulse. PPI_ratio_ does not take into account any additional information. Therefore, if you wanted to measure different aspects of the phenomenon of PPI, for instance, how the phenomenon of PPI changes across different startle sound levels, you would have to calculate many different PPI_ratio_ values (Fig. 1f, g).

**Fig. 2:**
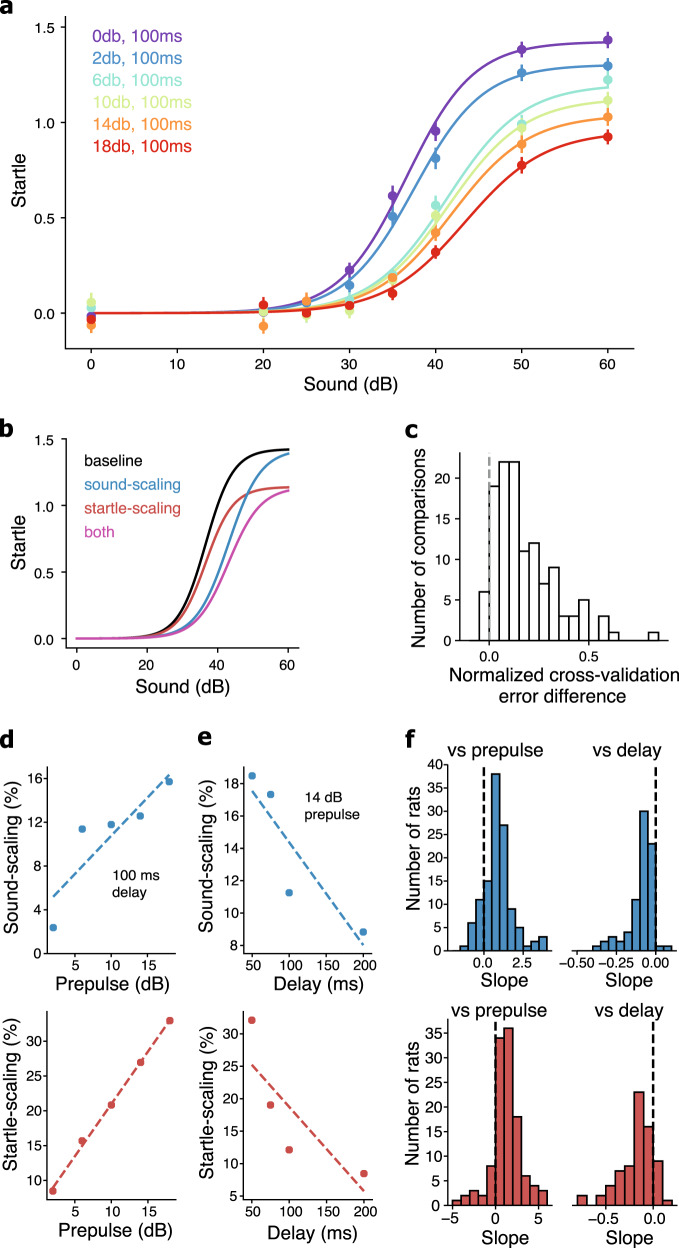
Startle- and sound scaling underlie the phenomenon of PPI. **a** Startle responses for one rat (same rat as Fig. 1c, f) across different startle sounds (*x*-axis) and prepulse sounds (colors) with a constant 100 ms delay. *x*-axis (and all subsequent dB references) indicates dB above background. Warmer colors indicate louder prepulse sounds. Bars indicate standard error of the mean. The normalized cross-validation (CV) error for this rat was 1.20; the median CV error across rats was 1.33 with an interquartile range of 0.26. **b** Diagram showing how a baseline startle curve (black) could be scaled via sound scaling (blue), startle scaling (red), or both startle- and sound scaling (magenta). **c** Distribution across all rats and experiments of the difference between the normalized cross-validation error of the model with both startle scaling and sound scaling, and the normalized cross-validation error of the model with only startle scaling. Positive error differences indicate that the two-parameter model had lower error, and the dotted vertical line shows error difference of 0. **d** Sound scaling versus prepulse sound (top) and startle scaling versus prepulse sound (bottom) for an example rat (the same rat as Fig. 2a) from an experiment that varied prepulse level. **e** Sound scaling versus delay (top) and startle scaling versus delay (bottom) for a different example rat from an experiment that varied delay. For **d** and **e**, dotted lines indicate linear regressions. **f** Distribution of linear regression slopes across all rats of sound scaling versus prepulse (top left), sound scaling versus delay (top right), startle scaling versus prepulse (bottom-left), and startle scaling versus delay (bottom right). Dotted lines show a slope of 0.

More fundamentally, understanding the phenomenon of PPI requires measuring the startle response across many different stimuli and asking how a prepulse changes the startle response under different conditions. We therefore measured the startle response of individual animals across a wide range of sound levels and across many different prepulse conditions (Fig. 2a). However, rather than computing many PPI_ratio_ values at all of those stimuli, we sought to understand formally how PPI changes the baseline startle response of an animal across the full range of startle sounds.

We are not aware of any formal model that underlies PPI_ratio_. A reasonable model would be a scaling of the startle by the prepulse, i.e., *m* = *m*_0_ + *αN*(*s*), where *m* is the movement in response to a startling sound, *m*_0_ is the baseline movement independent of sound, *α* is the startle scaling that occurs due to a prepulse, *s* is the sound level, and *N*(·) is a monotonically increasing function. With such a model, a straightforward derivation (see Supplementary Methods) shows that it is not possible for PPI_ratio_ to decrease with increasing startle sound levels, as long as *m*_0_ ≥ 0 and 0 < *α* ≤ 1. In other words, if the phenomenon of PPI represents just a downward scaling of the startle curve, then PPI_ratio_ cannot decrease with increasing startle sound levels. However, PPI_ratio_ does decrease with increasing startle sound levels (Fig. 1f, g), so the phenomenon of PPI must be more than just a downward scaling of the startle curve. To address this limitation, we developed a more comprehensive model-based analysis of the phenomenon of PPI.

We found that, for all of the prepulse conditions, the relationship between startle and sound level was well represented by a sigmoid function (Fig. 2a). We therefore chose a sigmoid as the monotonically increasing function *N*(·) (see Materials and Methods). We define the baseline startle curve as the sigmoid function that describes how an animal startles to the baseline prepulse condition, i.e., the condition with no prepulse sound. Our next step was to functionally describe how the baseline startle curve of an animal is modified by different prepulse conditions. Importantly, these modifications do not have to be purely a scaling along the startle axis (startle scaling), as other modifications such as a rightward scaling along the sound axis (sound scaling) could also describe aspects of the phenomenon of PPI (Fig. 2b).

We determined the specific functional form of these modifications to the baseline startle curve by revisiting the interpretation of PPI as one of sensory-motor gating [62]. Sensory-motor gating can occur in two fundamental ways: through modifying the movement that occurs in response to a sound or through modifying the processing of that sound. The first modification, startling a different amount in response to the same sound, could manifest through changes in bottom-up attention [63] or motor readiness. The second modification, processing the same sound differently, could manifest through sensory adaptation [64].

To disentangle these components, we introduce two parameters, *α*_*c*_ and *β*_*c*_, for each prepulse condition, *c*, which describe how the baseline startle curve is modified by the prepulse condition. Note that a prepulse condition is defined by two parameters: the prepulse sound level and the delay time between prepulse and startle sounds (see Materials and methods). For each prepulse condition, *c*, a given sound causes more or less startle as a function of *α*_*c*_, and a given sound is processed as more or less intense as a function of *β*_*c*_. Functionally, *α*_*c*_ and *β*_*c*_ scale the baseline startle curve along the startle and sound axes, respectively, and thereby represent the fundamental aspects of the phenomenon of PPI.

This yields the model $m = m_0 + \alpha _cN\left( {\beta _cs} \right)$, where *α*_*c*_ corresponds to startle scaling and *β*_*c*_ corresponds to sound scaling at prepulse condition *c*. This model contains both startle scaling and sound scaling, whereas, the model that underlies PPI_ratio_ contains only startle scaling (Eq. 2). Note, that with *β*_*c*_ < 1 the startle curve expands along the abscissa (sound axis) providing an increase in the difference between the startle curve with a prepulse when compared with without a prepulse. This scaling has the potential to help us understand the observed decrease in PPI_ratio_ with increasing startle sound (Fig. 1f, g). The difference between curves due to differences in sound scaling is maximal near the midpoint, and gets smaller as the curves approach their asymptotes (Fig. 2b), which would result in a decrease in PPI_ratio_ with increasing startle sound.

We found that our model containing two parameters that describe the phenomenon of PPI—with both startle- and sound scaling—was better than the model containing one parameter to describe the phenomenon of PPI, i.e., a model that implicitly underlies PPI_ratio_. The two-parameter model had lower cross-validated error than the one-parameter model in 118/124 (95.2%) of comparisons (Fig. 2c). Each rat contributed either one or two comparisons, depending on whether the rat was tested in one or two rounds of experimentation (see Materials and methods). The median normalized error of the two-parameter model was 0.13 lower than the median normalized error of the one-parameter model, meaning that our model with both startle scaling and sound scaling was a better fit to the data by ~13% of the standard error of the data points when compared with the model that implicitly underlies PPI_ratio_ with just startle scaling. This new model could also explain the known dependencies of PPI_ratio_ on prepulse condition [1, 65], and of self-reported sound intensity on prepulse condition [66, 67].

Prepulse conditions with greater-magnitude prepulse sounds and shorter delays produced greater scaling of the baseline startle curve (Fig. 2d, e). To quantify this effect, for each rat we fit lines to the PPI scaling parameters when compared with the prepulse sound intensity (Fig. 2d) and delay (Fig. 2e). We then analyzed the distribution of slopes across all rats, and we found that the distribution mean was significantly nonzero (*p* < 10^–8^, *t* test) (Fig. 2f). This indicates that both sound scaling and startle scaling increase with increased prepulse sound intensity and with decreased delay.

We then verified that the parameters obtained from an individual animal’s model fit were distinct from the parameters that best fit the other animals. We swapped each rat’s best-fit parameter set with the best-fit parameter set from the other rats in the same experiment, and recomputed the model-fitting error for the startle data for each rat (see Materials and methods) (Fig. S3a, left). Using the parameter sets from the different rats resulted in a median increase in model-fitting error of 0.179, which is more than four times the median best-fit error of 0.043 (*p* < 10^−269^, one-sided Wilcoxon signed-rank test) (Fig. S3a). This indicates that the model fits for individual animals yielded distinct parameters, allowing us to compare and interpret the parameter values for individual animals.

Importantly, our model greatly reduces the number of parameters required to understand PPI across a range of startle sounds. This is because our model has only two parameters—startle scaling and sound scaling—that describe how the animal’s entire baseline startle curve is modified. In contrast, the PPI_ratio_ represents only a point of the startle curve at a single startle sound level, so many different PPI_ratio_ values would be required to describe the animal’s PPI across a range of startle sounds.

Furthermore, everything described above is the case for different background sound levels, different animal ages, and different types of prepulse modifications (i.e., changing delay between the prepulse and startle sounds, and changing the intensity of the prepulse startle sounds) (see Supplementary Methods). Thus, the model represents a novel characterization of the phenomenon of PPI that is robust across many different experimental conditions.

### Analysis of group differences in model parameters

Up until this point, we have focused on an accurate understanding of the phenomenon of PPI for each individual animal in each prepulse condition. Each animal has three parameters describing its baseline startle curve, and an additional two parameters describing how each individual prepulse condition scales the baseline startle curve. Given that our model more accurately describes the phenomenon of PPI, we next sought to determine if it could provide a consistent description of the presence or absence of group differences. Therefore, for two cohorts of *Fmr1* KO and WT rats we evaluated whether these groups of animals differed in the five-dimensional space of these model parameters for each of the prepulse conditions.

Given that PPI can be affected by individual-animal factors, such as age [30–32], experience [43, 44], and strain [28, 29], we restricted our analyses of group differences to animals controlled for age and behavioral experience whose data were collected at roughly the same time. We first compared two cohorts of *Fmr1* KO (*n* = 18) and littermate WT male (*n* = 16) rats, along with a cohort composed of all WT males (*n* = 12) matched for age and experimental conditions with the first *Fmr1* KO cohort. The two *Fmr1* KO cohorts differed in age (Materials and methods) and experienced different prepulse conditions. These cohorts were therefore not directly compared within prepulse conditions.

**Fig. 3:**
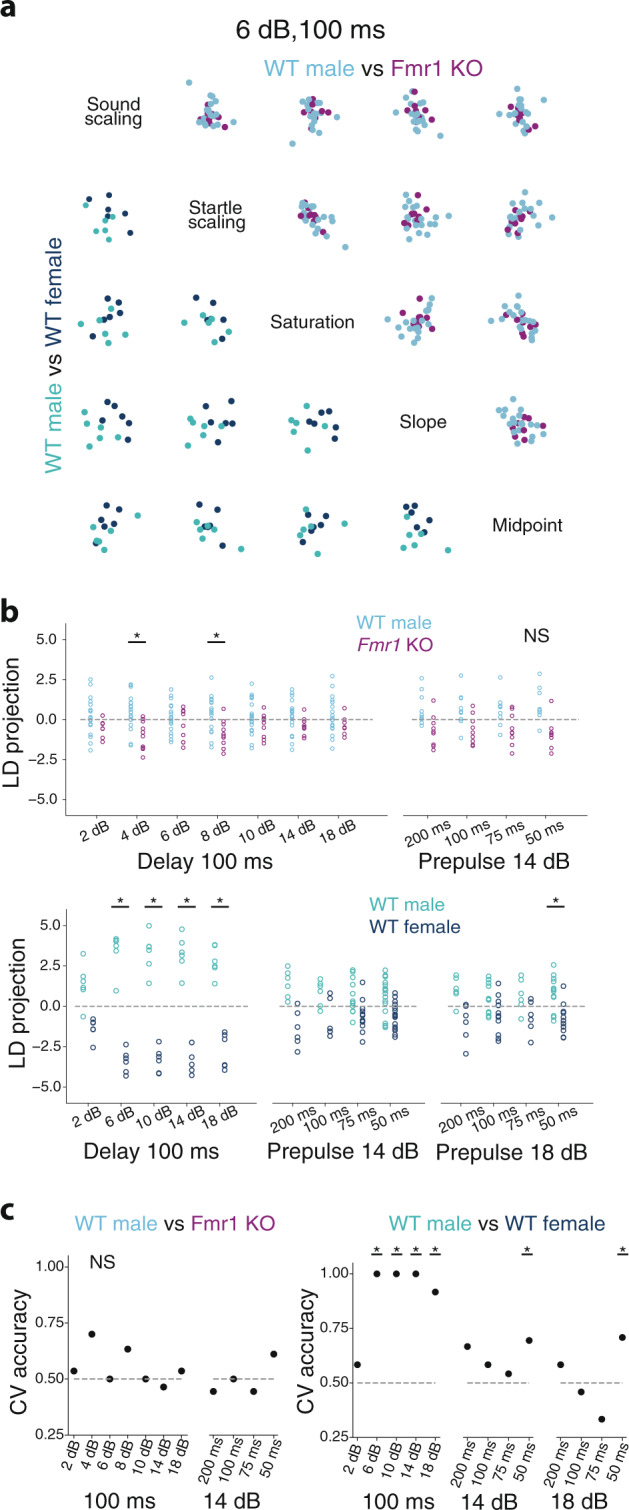
WT male and female rats, but not *Fmr1* KO and WT male rats, are linearly separable in their model parameters. **a** Pairwise scatter plots of the model parameters for WT male versus female rats (lower left), and *Fmr1* KO male versus WT male rats (upper right) for the prepulse condition with a 6-dB prepulse and 100-ms delay. All parameter values are mean-subtracted and standardized between 0 and 1. Main diagonal lists the five model parameters, such that each model parameter defines the *x*-axis and *y*-axis of the scatter plots in the same column and row, respectively. **b** Projections of the model parameters for *Fmr1* KO male versus WT male rats (top) and WT male versus WT female rats (bottom) onto the linear discriminate (LD), i.e., the vector orthogonal to the LDA hyperplane that best linearly separates the groups within each prepulse condition. Dashed horizontal lines indicate values that lie on the hyperplane that linearly separates the groups. Asterisks indicate prepulse conditions with *p* < 0.05 for the permutation test of total unsigned distance across all rats from the hyperplane separating the groups. **c** Leave-one-out cross-validation of LD prediction accuracy for *Fmr1* KO and WT male rats (left) and WT male versus female rats (right) for all prepulse conditions. Dashed horizontal lines indicate 50% prediction accuracy. Asterisks indicate prepulse conditions with *p* < 0.05 for the permutation test of leave-one-out cross-validation accuracy.

For each prepulse condition, we asked whether the model parameters distinguished between the groups. The five parameters for each animal in each group can be thought of as a point in a five-dimensional space (Fig. 3a), and we therefore used a linear discriminate analysis (LDA) to identify the hyperplane that best linearly separates the points associated with one group from those associated with the other. To quantify the group linear separability, we computed both the mean absolute distance of the points from each group to the LDA hyperplane and the accuracy of cross-validated predictions of group membership (see Materials and methods).

For the *Fmr1* KO male and WT male groups, we found only 2/11 prepulse conditions where the mean absolute distance from the LDA hyperplane was significantly large (*p* < 0.05, permutation test) (Fig. 3b, top), which is not significant after controlling for multiple comparisons (*p* > 0.10, bootstrapped ratio test). We also found that there were no prepulse conditions where the cross-validated classification was significantly greater than chance (*p* > 0.09, permutation test) (Fig. 3c, left). Thus, the *Fmr1* KO and WT male rats were not linearly separable in their model parameters when compared within prepulse conditions.

We note, however, that it is still possible that *Fmr1* KO and WT rats could be linearly separable in the high-dimensional space that includes all of the model parameters across all of the prepulse conditions. The above-described experiments were not designed for that analysis, since different cohorts of animals were subjected to different prepulse conditions, something that was done to generalize the model across a range of conditions. Future experiments will be needed to rule out that possibility.

Importantly, the apparent lack of group differences was not a result of the additional complexity of our model: the same approach yielded clear differences between male and female rates, consistent with previous reports [33, 34, 36]. Using the same methodology, we compared two cohorts of animals composed of WT female (*n* = 12) and male (*n* = 12) rats. We computed an LDA on the model parameters across all rats in the WT male and WT female groups. We found that the animals’ mean absolute distance from the LDA hyperplane was significantly large in 5/13 prepulse conditions (*p* < 0.05, permutation test) (Fig. 3b, bottom), which is significant after controlling for multiple comparisons (*p* < 10^−3^, bootstrapped ratio test). Furthermore, the cross-validated classification accuracy was significantly greater than chance in 6/13 prepulse conditions (*p* < 0.05, permutation test) (Fig. 3c, right), which is significant after a control for multiple comparisons (*p* < 10^−4^, bootstrapped ratio test). Thus, the WT female and WT male rats were linearly separable in their model parameters, and our more accurate and complex model is capable of detecting group differences when they are present.

### PPI covaries with the baseline startle curve

The above results, using LDA, represent a way to identify group differences in the startle response within a prepulse condition, but they do not establish differences in PPI, as the LDA was carried out on both the parameters for the baseline startle response and the PPI scaling parameters. We did not directly look for group differences in the PPI scaling parameters (startle scaling and sound scaling), because we suspected that the PPI scaling parameters could be correlated with the baseline startle curve, given that PPI_ratio_ has been reported to covary with the baseline startle response [45]. If PPI covaries with the baseline startle curve, then correctly interpreting group differences in PPI would require taking the baseline startle into account.

**Fig. 4:**
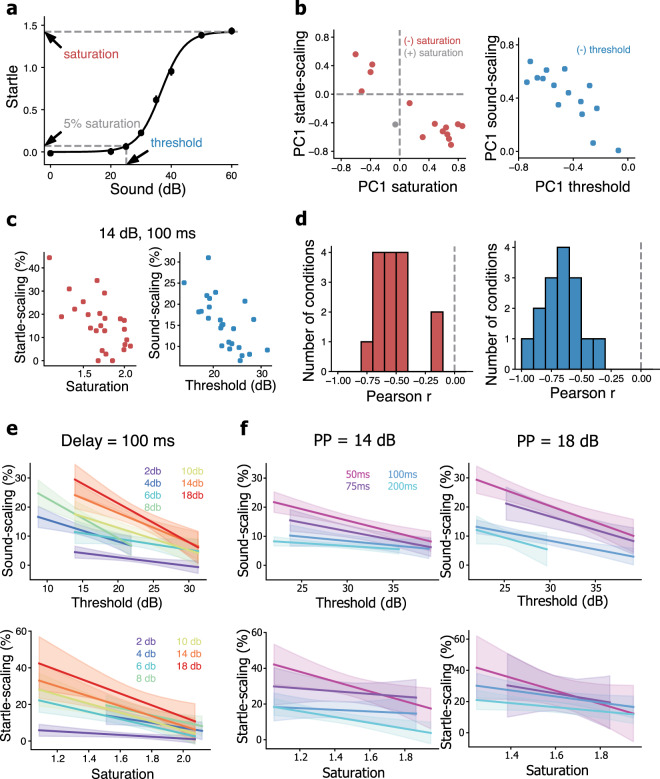
Sound- and startle scaling covary with baseline startle curve. **a** Example baseline startle curve for the same animal from Fig. 2a. Arrows and dashed lines indicate the saturation, defined as the asymptotic maximum of the sigmoid function, and the threshold, defined as the sound at which the curve reaches 5% of saturation. Solid line shows fit of the model to the data. **b** Scatter plot of PC1 startle-scaling weight versus PC1 saturation weight (left) and PC1 sound-scaling weight versus PC1 threshold weight (right) across all prepulse conditions. Dashed vertical and horizontal lines indicate PC1 weights of 0. Red points indicate prepulse conditions with opposite direction PC1 weights between startle scaling and saturation. Blue points indicate prepulse conditions with opposite direction PC1 weights between sound scaling and threshold. **c** Example scatter plots of startle scaling versus saturation (left, *r*^2^ = 0.40) and sound scaling versus threshold (right, *r*^2^ = 0.44). For both plots the prepulse sound was 14 dB and the delay was 100 ms. **d** Distribution of Pearson’s *r* values for all prepulse conditions of startle scaling versus baseline saturation (left) and sound scaling versus baseline threshold (right). Dotted vertical line shows *r* value of 0. **e** Linear regressions of sound scaling versus baseline threshold (top) and startle scaling versus baseline saturation (bottom) at different prepulse sound levels. Warmer colors indicate louder prepulse sounds. **f** Linear regressions of sound scaling versus baseline threshold (top) and startle scaling versus baseline saturation (bottom) at different delays. Warmer colors indicate shorter delays. For **e** and **f**, the shaded area around lines indicates 95% confidence intervals of the regression.

We therefore asked whether PPI startle scaling and sound scaling covary with features of the baseline startle curve. To identify if there are correlations between the parameters of the model for individual animals, we ran principal component analysis (PCA) across all of the WT male animals in each prepulse condition. To simplify the information provided by PCA, we combined the two parameters from the baseline startle response curve that relate to the sound axis (midpoint and slope) into a single value: the threshold (Fig. 4a). Therefore, for each prepulse condition, this left us with four parameters: a single-baseline parameter describing the startle axis (saturation), a single-baseline parameter describing the sound axis (threshold), a parameter describing scaling of the startle axis (startle scaling), and a parameter describing the scaling of the sound axis (sound scaling).

Consistent with there being correlations within these four parameters, the first-principal component (PC1) explained 42–69% (mean 52%) of the variance and was significant in 8/15 prepulse conditions (*p* < 0.05, permutation test) (Fig. S4). This is a significant number of prepulse conditions after controlling for multiple comparisons (*P* < 10^−6^, bootstrapped ratio test). Strikingly, in 14/15 prepulse conditions, the PC1 startle-scaling weight was in the opposite direction of the saturation weight (Fig. 4b, left), and in all 15 prepulse conditions, the sound-scaling weight was in the opposite direction of the threshold weight (Fig. 4b, right).

These opposing signs within the first-principal component highlight a relationship between sound scaling and threshold, and separately, between startle scaling and saturation. Indeed, within each prepulse condition, we found that startle scaling was negatively correlated with the saturation level of the baseline startle curve across all of the WT male rats (Fig. 4c, d). Animals with higher startle saturation, i.e., higher maximum startle, tend to have less startle scaling. The mean Pearson’s *r* was −0.51 ± 0.04, and the *r*^2^ values ranged from 0.02 to 0.62 (Fig. 4d), meaning that the startle saturation accounted for up to 62% of the variance of the startle scaling across rats within prepulse conditions. This correlation was significant in 10/15 prepulse conditions (*p* < 0.05, Pearson’s correlation).

Similarly, within each prepulse condition, we found that sound scaling was negatively correlated with startle threshold of the baseline startle curve across all of the WT male rats (Fig. 4c, d). Animals with higher startle thresholds tend to have less sound scaling. The mean Pearson’s *r* was −0.65 ± 0.04, and the *r*^2^ values ranged from 0.13 to 0.83 (Fig. 4d), meaning that the startle threshold accounted for up to 83% of the variance of the sound-scaling across rats within prepulse conditions. This correlation was significant in 11/15 prepulse conditions (*p* < 0.05, Pearson’s correlation).

These correlations were not the result of imprecision in the estimates of the model parameters for individual animals. In addition to assessing the significance of the observed correlations, as described above, we also evaluated the robustness of the correlations to imprecision in the estimates of the various parameters of the model (see Materials and methods). The majority of the observed correlations were robust to resampling the correlations from the range of parameters that would occur due to noise in the data (Fig. S3c). Thus, the estimation precision of the parameters for individual rats was sufficient to identify real correlations across rats between the PPI scaling and baseline startle parameters.

Furthermore, the correlations were not a result of compensations between the parameters of the model that were only exposed due to noise in the data. For example, it could have been possible that there was a fixed relationship between the different parameters of the model, such that if there was a decrease in the saturation, then there had to be a corresponding change in the startle scaling. This could manifest as the observed correlations in the parameters that would come about just due to noise in the data. We ruled out this possibility by measuring the relationship between the different parameters of the model that would occur just due to noise in the data (see Materials and methods).

All of the observed across-animal correlations between the startle scaling and saturation (Fig. 4d) were stronger than the randomly generated within-animal correlations, indicating that the correlations that we observed between the startle scaling and saturation cannot be due to compensations between parameters of the model (Fig. S3d). In fact, the correlations that would occur due to compensations between the parameters go in the opposite direction from the observed correlations.

The correlations that occur between sound scaling and the threshold due to compensation between the parameters go in the same direction as our observed correlations. However, in 13/15 prepulse conditions, the observed across-animal correlations between the sound scaling and the threshold were larger than the 75th percentile of the within-animal correlations just due to parameter compensation. Moreover, in 11/15 conditions the correlations in the data was outside the range of the outliers, defined as the 75th percentile minus 1.5 times the interquartile range, i.e., the lower whisker (Fig. S3d). This indicates that across the entire set of experiments, the observed correlations between the scaling parameters and the baseline startle curve across animals cannot be explained by compensation between the parameters of the model.

These findings establish the presence of strong relationships between the baseline startle response and PPI. To model those relationships, we computed linear regressions for startle scaling as a function of baseline saturation, and for sound scaling as a function of baseline threshold (Fig. 4e, f). The correlation between the PPI scaling and baseline parameters showed a range of values (Fig. 4d) where the slope of the regressions increased with increasing prepulse sound level and with shortened delay (Fig. 4e, f and S5). Thus, the phenomenon of PPI is both a function of prepulse condition and of the baseline startle curve, and differences in PPI can only be interpreted with respect to the baseline startle parameters of individual animals.

### Analysis of PPI group differences

**Fig. 5:**
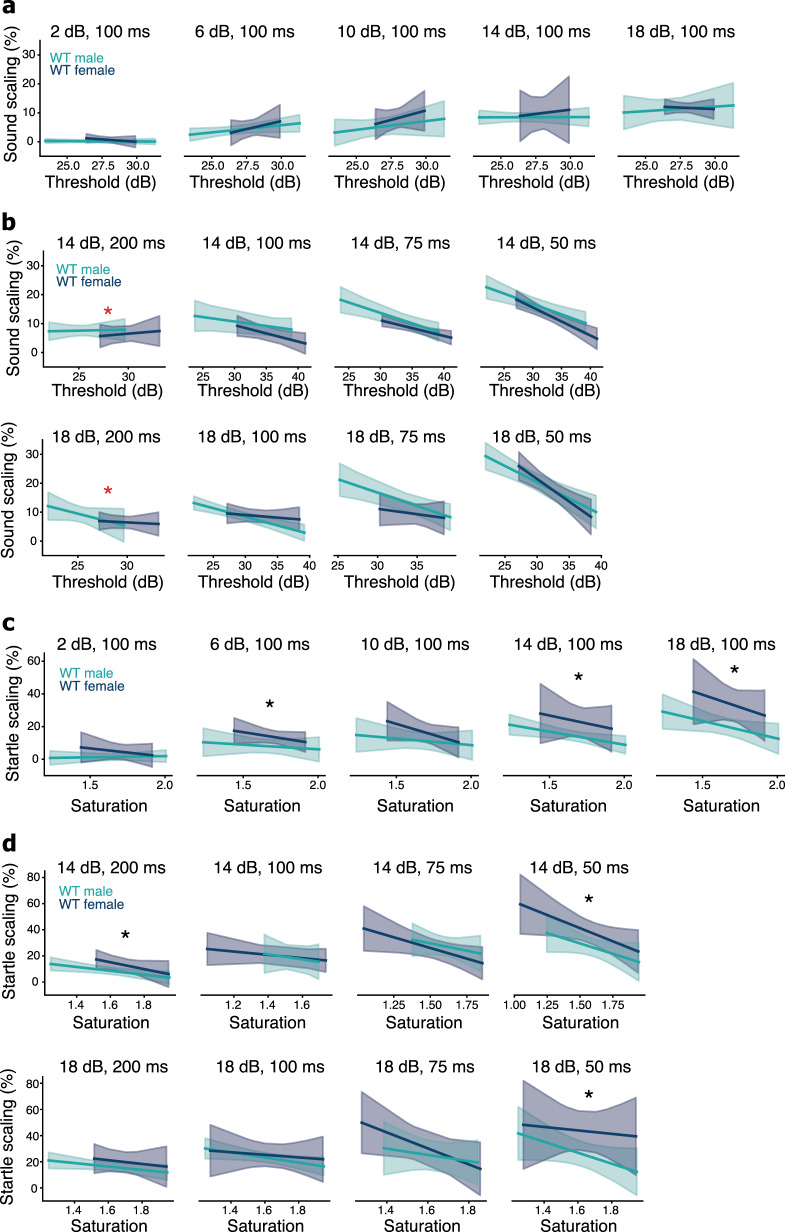
Less startle scaling in WT male than WT female rats. **a** Linear regressions of sound scaling versus baseline threshold for WT male (green) and female (gray) rats from the single experiment that varied prepulse sound level with a constant 100-ms delay. Subplots show increasing prepulse sound level from left to right. **b** Linear regressions of sound scaling versus baseline threshold for WT male and WT female rats from the three experiments that varied delay with a 14 dB (top) or 18 dB (bottom) prepulse. Subplots show decreasing delay from left to right. Red asterisks indicate prepulse conditions with a group difference in baseline threshold (*p* < 0.05, *t* test). This was not significant after controlling for multiple comparisons (*p* > 0.05, bootstrap ratio test), but these two prepulse conditions were excluded from ANCOVAs. **c** Linear regressions of startle scaling versus baseline saturation for WT male and WT female rats from the single experiment that varied the prepulse sound level with a constant 100 ms delay. Subplots show increasing prepulse sound level from left to right. **d** Linear regressions of startle scaling versus baseline saturation for WT male and female rats from the three experiments that varied delay with a 14- (top) or 18-dB (bottom) prepulse. Subplots show decreasing delay from left to right. For **c**, **d** black asterisks indicate prepulse conditions where WT male rats had lower startle scaling than WT female rats (*p* < 0.05, ANCOVA). For **a**, **b**, **c**, and **d** there were no baseline-by-group interactions (*p* > 0.05, ANCOVA). In total, WT male rats had lower startle scaling than WT female rats in 6/13 prepulse conditions, which is significant after multiple comparisons (*p* < 0.05, bootstrap ratio test).

These findings indicate that adjusting for baseline covariates is important when comparing groups for differences in PPI startle- and sound scaling. Therefore, for each prepulse condition, we computed two linear regression models across all of the WT male rats and, separately, across all of the WT female rats. These linear models describe the two correlations between the scaling and baseline startle parameters: sound scaling versus baseline threshold (Fig. 5a, b) and startle scaling versus baseline saturation (Fig. 5c, d). We then computed an ANCOVA with interaction term for each prepulse condition.

After confirming that there was no baseline-by-group interactions (*p* > 0.05), we re-ran the ANCOVAs without an interaction term. We found no prepulse conditions with a group difference in baseline saturation (*p* > 0.05, *t* test), but we did find two prepulse conditions with a group difference in baseline threshold (Fig. 5b) (*p* < 0.05, *t* test). However, a control for multiple comparisons reveals 2/13 significant prepulse conditions to be insignificant (*p* > 0.1, bootstrapped ratio test), and the exclusion of those two prepulse conditions did not affect the results. Thus, the differences between the groups could not be explained by a difference in baseline parameters.

We then considered the effects of group on PPI as measured by the startle- and sound-scaling parameters. We found that WT female rats had greater startle scaling than WT male rats (*p* < 0.05, ANCOVA) in 6/13 prepulse conditions, which is significant after controlling for multiple comparisons (*p* < 10^−4^, bootstrapped ratio test). In contrast, we found no differences between WT female and male rats in sound scaling at any prepulse condition (*p* > 0.05, ANCOVA). As a confirmation, running LDA on the features of these models—startle scaling, sound scaling, saturation, and threshold—results in 7/13 significant prepulse conditions (*p* < 0.05, permutation test) (data not shown), including the same six significant prepulse conditions as found with ANCOVA.

Finally, to confirm our previous results, we carried out the same analysis on the *Fmr1* KO and WT male rats. There were no prepulse conditions where the *Fmr1* KO male rats differed from WT male rats in sound scaling (*p* > 0.05, ANCOVA) (Fig. S6). There was one condition where *Fmr1* KO rats had lower startle scaling than WT male rats (*p* = 0.02, ANCOVA), but this was not significant after a control for multiple comparisons (*p* > 0.4, bootstrapped ratio test) (Fig. S6). There were also no differences in either baseline saturation or baseline threshold (*p* > 0.05, *t* test). Thus, we were unable to detect differences in PPI between *Fmr1* KO and WT male rats, confirming our finding that *Fmr1* KO male rats were not linearly separable from the WT male rats in their model parameters (Fig. 3b, c). These results in the *Fmr1* KO rat provide a reliable approach that could be used to clarify the inconsistent PPI results with *Fmr1* KO mice and humans with FXS.

## Discussion

We found inconsistent PPI_ratio_ results in *Fmr1* KO rats at different startle sound levels within cohorts, and at similar startle sound levels between cohorts (Fig. 1a, b), extending the inconsistent results seen in the *Fmr1* KO mouse literature [39, 49–56] to a different species. Furthermore, we confirmed that the acoustic startle response is better described by a log-normal than a Gaussian distribution (Fig. 1c–e), and that the PPI_ratio_ changes across startle sound levels [48] (Fig. 1f, g). These results reveal important limitations of the traditional PPI methodology.

To address these limitations, we developed a new model of PPI (Fig. 2a), which describes how a prepulse sound scales the baseline startle curve along both the startle and sound axes (Fig. 2b). We found that our model was a consistently better description of the data for individual animals than the implicit model underlying the PPI_ratio_ metric (Fig. 2c). This shows that the phenomenon of PPI consists of both a reduction in the startle response (startle scaling) and a reduction in sound intensity (sound scaling). We then found that *Fmr1* KO male rats were not linearly separable from WT controls in their model parameters. In contrast, we found that WT male and female rats are linearly separable in their model parameters (Fig. 3).

Seeking to explain these differences, we found that startle- and sound scaling were correlated with the baseline startle response curve across animals within prepulse conditions (Fig. 4b–d). Taking this into account, we analyzed group differences in startle scaling and sound scaling by fitting linear models to the scaling versus baseline data. We found no difference in PPI between *Fmr1* KO and WT rats (Fig. S6). We did, however, find that WT female rats showed greater PPI startle scaling than WT male rats (Fig. 5c, d). These findings were robust to changes in startle sound level, and they were reliable across different cohorts of animals.

### Benefits of a new model of PPI

The phenomenon of PPI exists independent of the PPI_ratio_ metric or any other model used to describe it. Fundamentally, animals tend to startle less when a startling stimulus is preceded by a prepulse, compared with when a startling stimulus is presented alone. But what is the functional form of this phenomenon, and how does it depend on the stimulus and individual differences between animals? The usefulness of PPI in neuroscience and psychiatry depends on our ability to understand the phenomenon itself, and this in turn depends on the metrics and models used to describe the phenomenon.

Here, we present a novel model that disentangles two components underlying PPI: sound scaling and startle scaling. In contrast, the model that implicitly underlies the PPI_ratio_ metric does not describe scaling of the startle sound. Previous work has observed changes in perceived sound after a prepulse [66–68], but this has been conceptualized as a separate phenomenon from PPI, often measured using self-report scales. Our model unifies startle and sound scaling, revealing them to be two components of the phenomenon of PPI, both of which can be observed in the acoustic startle data.

Furthermore, both sound scaling and startle scaling are biologically interpretable. Sound scaling could manifest through rapid sensory adaptation in auditory hair cells [69] or higher auditory pathways [70], while startle scaling could manifest through changes in bottom-up attention or other cognitive or motor factors [63]. A formal model that separately parameterizes sound scaling and startle scaling allows for a principled deconstruction of the behavioral neurobiology of PPI. In our case, we were able to quantify how much of each parameter contributed to PPI in individual animals (Fig. 2), how they covaried with the baseline startle (Fig. 4), and how they compared between groups (Figs. 5 and S6).

If PPI is to be a useful biomarker of disease [21] or a predictor of treatment outcomes [22], then at a minimum we need to describe the core behavioral features of the phenomenon using a reproducible methodology. By showing that startle- and sound scaling underlie the phenomenon of PPI, our model provides such a methodology.

### Assumptions and limitations of the model

One of the challenges that we faced in deconstructing the current way in which the phenomenon of PPI is measured was in disentangling the many assumptions that underlie the current metric used to describe the phenomenon. Therefore, we feel it crucial to lay out the assumptions, and the potential limitations of those assumptions, that underlie our proposed model. We used many repeats of each stimulus, which is important given the high variability of the startle response between trials. The alternative—using a small number of trial repeats—suffers from a potentially inaccurate representation of the underlying startle distribution.

However, combining data across many trials assumes that the startle response is relatively stable across trials. Furthermore, by randomly presenting dozens of different stimuli conditions within a session, we assume that there are minimal between-trial dependencies. Although PPI is not thought to habituate across trials [7, 25], we did observe small increases in startle scaling from the first to the second halves of the experiments (see Supplementary Methods). This is consistent with prior results showing that PPI_ratio_ increases with repeated testing [59]. Nevertheless, we chose to combine data across trials within an experiment, allowing us to develop an accurate statistical representation of the acoustic startle response and a reliable static model of PPI. Further experiments could incorporate a more dynamic picture into the interpretation of the phenomenon of PPI, using the model proposed here as a foundation.

Accurately fitting the model required measuring the startle response across a full range of startle sounds (see Supplementary Methods), the loudest of which can be louder than is typical in PPI experiments. This raises a concern that hearing loss could have influenced our results, particularly for the older animals. If it were the case that hearing loss occurred due to louder sounds, we would expect to see greater changes in the baseline startle curve between the first and second halves of experiments with louder absolute sounds and older animals, compared with experiments with lower absolute sound levels and younger animals. We did not see such changes; we found no differences in the magnitude of changes in any of the baseline startle parameters for experiments with louder sounds and older animals, compared with experiments with weaker sounds and younger animals (see Supplementary Methods). Nevertheless, in both types of experiments, we did observe small but significant changes that are more likely explained by habituation, or other dynamics not directly attributable to the maximal sound level.

As we have justified (Fig. 1c–e), the basis for our measurement of the startle response rests upon the assumption of the lognormality of the data. Skewed data in complex biological systems is a common finding [71], reflecting interactions in complex systems such as the brain. However, further experimentation could expose that the startle distribution could be more accurately described by more complex distributions, such as variants of the gamma distribution or combinations of several distributions. For example, it is possible that a scalar measurement of startle magnitude only makes sense in a subset of “true startle” events, as distinct from “no startle” events. If so, it could be useful to consider a probability of startle in addition to the magnitude of startle.

We also introduced a new axis along which PPI changes an animal’s response to a startle sound: by scaling the processing of sound itself. To start with the simplest possible model, we assumed that the sound scaling occurred through a single parameter that multiplied the sound axis. This relatively simple addition to the model provided clear explanatory power, but a complete description of the phenomenon could include an additional parameter that shifts the sound axis. We also chose to use a reduced set of model features to help adjust for baseline covariates by substituting the baseline threshold for the slope and midpoint parameters. This choice provides a highly interpretable parameter (baseline threshold), and the first principle component of these four features explains about the same amount of the variance in the data (mean 48%) as using all five model parameters (mean 52%). Nonetheless, using all five model parameters, or features other than the threshold, could also likely describe the data. More broadly, we note that a full description of the phenomenon of PPI would be a high-dimensional surface that describes how an animal startles under all different stimuli [61]. Modeling this surface will require manipulating multiple stimulus variables, e.g., prepulse level and delay, within the same animals, something we did not do in our experiments.

Our results make it clear that there is more to PPI than just a ratio of the startle with a prepulse to the startle without a prepulse. PPI is a complex phenomenon that depends on many features of the stimulus, and which shows high variability between individual animals. In spite of, or perhaps because of this complexity, PPI could be a useful methodology for generating mechanistic insights into neuropsychiatric disease, as evidenced by the extensive literature linking PPI_ratio_ to schizophrenia [4–9] and other disorders [10–20]. As a step toward that goal, our analytical model allows for a deconstruction of the underlying structure of PPI, which in turn enables robust and replicable studies of the neural circuits underlying PPI, and how those circuits vary among individuals in the context of disease.

## Supplementary information

Supplemental Material

## Data Availability

See Supplementary Methods for a description of the protocol for the full analyses. Analyses were done in python, and the code to fit the PPI model is available at https://github.com/angevineMiller/ppi_model.
