# Supplementary material for: Robust and replicable measurement for prepulse inhibition of the acoustic startle response
Source: Mol Psychiatry. 2020 Mar 6;26(6):1909–27. doi: 10.1038/s41380-020-0703-y (PMC7483293; doi:10.1038/s41380-020-0703-y)

Supplementary Methods:

Group comparisons with classical PPI methodology

We computed the PPI_{ratio} on the *Fmr1* KO versus WT male comparison using the gain-normalized data before taking the log of the startle response. There were a total of 9 different startle sound levels between 20 – 60 dB above background (Supplementary Table 1). The second *Fmr1* cohort was delay-varying, which we always analyze separately from prepulse-varying cohorts. Therefore, in total, we ran 16 different 2-way ANOVAs on PPI ratio versus group and prepulse sound level or delay. Each comparison had between 2 – 7 different prepulse levels or delays (mean 5). We found 3/16 comparisons in which *Fmr1* KO rats had increased PPI compared to WT male rats ($p < 0.05$, 2-way ANOVA), which is more than we expect by chance ($p = 0.042$, bootstrapped ratio test). Furthermore, all three comparisons held up to Bonferroni correction ($p < 0.05/18$). Based on this result, one could have concluded that *Fmr1* KO rats showed increased PPI compared to WT male rats. This could also be explained by group differences in the baseline startle response (Fig. 4) or by other aspects specific to the classical PPI methodology or the PPI_{ratio} metric (Fig. 1). However, given the analyses described in the rest of this paper and the better accuracy of our model in describing the data, we chose to not pursue these analyses further.

Accurate model fit requires full coverage of startle curves

We chose a range of startle sounds that elicited startle responses covering the animals' full startle response functions in order to accurately fit the model to the data (Fig. 2a). In particular, the louder startle sounds were necessary for most of the animals to startle close to their saturation values. The average fraction of startle saturation was 100% to the loudest sound but only 93% and 72% to the second and third loudest sounds, respectively. Note that it is possible for an animal to startle at greater than 100% startle saturation when the data point is above the model curve (Fig. 2a). Next, we tried fitting the model without including data from the loudest startle sound, and another time without including the loudest two startle sounds. We then computed the average model fitting error using all of the data. The average RMSE was over 2 times higher if we exclude the loudest startle sound during fitting and over 4 times higher if we exclude the loudest two sounds.

Unbounded scaling parameters do not improve model accuracy

We fit the model with the startle-scaling or sound-scaling parameters, α_c and β_c, bounded between 0 (100% scaling) and 1 (0% scaling) to minimize the potential for compensation of these two parameters. However, α_c and β_c greater than 1 (i.e. negative scaling) could also represent prepulse facilitation (PPF) or heightened sensory arousal. Therefore, we separately tried fitting the model without any bounds on these parameters.

For the unbounded fits, we found that 21/488 (4.3%) rat-conditions had negative startle-scaling ($\alpha_c > 1$) and 10/488 (2.0%) rat-conditions had negative sound-scaling ($\beta_c > 1$). While the unbounded model had an ~1% lower median RMSE, this did not hold up to cross-validation. Rather, the normalized cross-validation error was no different in the two models ($p > 0.05$, t-test), and the bounded model actually had a lower normalized cross-validation error in 65/124 (52.4%) of rat-experiments. To better understand the fits, we examined the rat-conditions with negative scaling more closely. None of these rat-conditions were convincing examples of PPF or heightened arousal, but we observed two general phenomena that could explain the few instances

of negative scaling: 1) Some rat-conditions had a small negative sound-scaling to a very weak prepulse condition, such as the 2 dB prepulse sound. This was likely due to overfitting to the noise for conditions with little or no actual PPI. This could explain why the unbounded model was no better than the bounded model after cross-validation. And 2) some rat-conditions had a large negative startle-scaling to a strong prepulse condition, such as the 18 dB prepulse sound. In fact, these strong prepulse conditions had so much PPI that we did not have many data points close to startle saturation, a further indication of the presence of sound-scaling. Without a well-defined saturation point, the model was free to converge on less biologically plausible startle-scaling values to compensate for other parts of the fit. In this case, the model appeared to be using a negative startle-scaling to indirectly increase the slope of the prepulse curve to better fit the rising part of the curve. For these reasons, we chose to stick with the more interpretable model with α_c and β_c bounded between 0 and 1.

Standard $\text{PPI}_{\text{ratio}}$ **assumptions cannot describe the phenomenon of PPI**

Here we derive that the $\text{PPI}_{\text{ratio}}$ metric can never decrease as a function of increasing startle sound if PPI is just due to a scaling of the startle response, under the assumption that the acoustic startle response is well captured by any monotonically increasing function.

Assume for the sake of contradiction that $\text{PPI}_{\text{ratio}}(s_1) > \text{PPI}_{\text{ratio}}(s_2)$ for some startle sounds $s_1 < s_2$. By the definition of $\text{PPI}_{\text{ratio}}$ (Materials & Methods Eq. 1), this gives:

$$\frac{m_b(s_1) - m_p(s_1)}{m_b(s_1)} > \frac{m_b(s_2) - m_p(s_2)}{m_b(s_2)} \qquad \text{Eq. S1}$$

where m_b is the average startle response to the startle sound alone, i.e. the baseline startle response, and m_p is the average startle response of the animal to the startle response preceded by the prepulse sound.

Let $N(s)$ be any monotonically increasing, strictly positive function of the sound that well-describes the acoustic startle response. Furthermore, let m_0 be a constant non-negative y-offset due to baseline movement. Therefore, $m_b(s_1) = m_0 + N(s_1)$ is the baseline startle response at sound s_1, and $m_p(s_1) = m_0 + \alpha N(s_1)$ is the startle response at sound s_1 following a prepulse, where α is the startle-scaling parameter. Plugging this into Eq. S1, this gives us:

$$\frac{m_0 + N(s_1) - m_0 - \alpha N(s_1)}{m_0 + N(s_1)} > \frac{m_0 + N(s_2) - m_0 - \alpha N(s_2)}{m_0 + N(s_2)}$$

The left side of the equation calculates the $\text{PPI}_{\text{ratio}}$ metric at sound s_1 and the right side of the equation calculates $\text{PPI}_{\text{ratio}}$ metric at sound s_2. We then subtract off the right side of the equation and cancel out the baseline movements (m_0) in the numerators such that:

$$\frac{N(s_1) - \alpha N(s_1)}{m_0 + N(s_1)} - \frac{N(s_2) - \alpha N(s_2)}{m_0 + N(s_2)} > 0$$

If $\alpha \neq 1$, then we can then factor out $1 - \alpha$ from both numerators and divide them out of the equation. Furthermore, by definition, PPI implies that $1 - \alpha > 0$, so we maintain the direction of the inequality, giving:

$$\frac{N(s_1)}{m_0 + N(s_1)} - \frac{N(s_2)}{m_0 + N(s_2)} > 0$$

We then combine the fractions and simplify the numerator to get:

$$\frac{m_0 N(s_1) - m_0 N(s_2)}{\big(m_0 + N(s_1)\big)\big(m_0 + N(s_2)\big)} > 0$$

Assuming that the baseline movement, m_0, is strictly positive, so we can factor m_0 out from the numerator:

$$\frac{N(s_1) - N(s_2)}{\left(m_0 + N(s_1)\right)\left(m_0 + N(s_2)\right)} > 0$$

This cannot be true given our definition of $N(s)$ as a monotonically increasing function that is always greater than zero. Thus, we have reached a contradiction, and Eq. S1 must be false.

Thus, the $\text{PPI}_{\text{ratio}}$ metric can never decrease if PPI is just due to scaling the startle response, under the assumption that the acoustic startle response is well captured by any monotonically increasing function that is always positive.

Protocol for measuring and fitting sound and startle-scaling PPI model.

1) Measure the acoustic startle response at many different stimuli.
    a) Vary the startle sound level across the full range of values over which the startle response changes. For example, we varied the startle sound level between 0 – 60 dB above background, as this was the range over which our rats' startle responses varied from zero to maximum startle.
    b) Vary the prepulse sound level and/or the delay time across the range of values over which PPI changes as a function of that parameter. For example, we varied the prepulse sound between 0 – 18 dB above background and the delay between 50 – 200 ms. To get a measurement of PPI, it is only necessary to pick a single prepulse sound level and delay. We sought to understand a large swath of the phenomenon and therefore utilized many different prepulse sound levels and delays.
    c) For each stimulus—i.e. combination of prepulse sound, delay time, and startle sound—collect data from at least 50-100 trial repeats for every animal tested.
    d) For each trial, normalize the raw accelerometer data by a baseline accelerometer measure, e.g. by the data from times prior to the presentation of any stimulus (Fig. S2a&b).
    e) Take the $\log_{10}$ of all of the normalized accelerometer data.
    f) For each trial, find the maximum value of the log normalized data in a 100 ms window following the presentation of the startle sound.
2) Compute the average startle at each stimulus
    a) For each rat, find the mean and standard error of the trial maxima from 1f at each stimulus.
    b) For every mean startle value from 2a, subtract the mean value across all of the control stimuli, i.e. the stimuli with startle sound level 0 dB above background across all prepulse conditions.
    c) We define the resulting values as the startle to a given stimulus for an animal, and we can plot these values as startle response versus startle sound (Fig. 2a).
3) Fit the PPI model to the average startle data for each rat. Python code to implement this step can be found at https://github.com/angevineMiller/ppi_model.
    a) Implement a sigmoid function with startle-scaling and sound-scaling parameters (Materials and Methods Eq. 3&4). This function should accept 5 parameters for each prepulse condition: $m_{\max}$, s_0, and r for the baseline sigmoid and α_c and β_c for the scaling of the baseline sigmoid due to a prepulse condition, c. Note that α_c

and β_c, but not the baseline sigmoid parameters, change across different prepulse conditions within the same animal.

b) Implement an objective function that computes the *total* RMSE across *every* stimulus between the average startle response data from 2c and the model predictions at those stimuli. This RMSE is computed under a choice of model parameters for every prepulse condition (i.e. $m_{\max}$, s_0, r, α_c, and β_c for all prepulse conditions, c). In Figure 2a, this can be seen as the total error across all of the data points and their corresponding model curves of the same color.

c) Use a minimization algorithm (e.g. Scipy.optimize) to find the optimal model parameters that minimize the objective function against the average startle data for each individual rat. Initial conditions for the scaling parameters can be set to no scaling. Initial conditions for the baseline sigmoid can be set to anything that you think will optimize the chances of converging on the best fit (we chose the parameters that best fit the sigmoid to the startle values with no prepulse).

4) Evaluate group differences in the model parameters
   a) Standardize the parameters to all range between 0 – 1 and subtract the means.
   b) For each prepulse condition, run a linear classifier such as linear discriminate analysis (LDA).
   c) Compute the mean absolute (unsigned) distance from the linear discriminate hyperplane.
   d) Compute LDA classification accuracy using leave-one-out cross-validation.
   e) Report group separability if the mean absolute distance and the cross-validated classification accuracy are significantly great from permutation tests on the group labels.

5) Find baseline threshold and saturation for each animal
   a) The baseline saturation is defined as $m_{\max}$ of the baseline sigmoid for a given animal.
   b) Compute the baseline threshold, defined as the startle sound level at which an animal's baseline startle curve reaches 5% of $m_{\max}$.

6) Evaluate group differences in PPI
   a) For each prepulse condition, fit two linear models per group: one for sound-scaling versus baseline threshold and one for startle-scaling versus baseline saturation, and plot these with 95% confidence intervals (Fig. 5&S6).
   b) For each prepulse condition, check for group difference in the baseline parameter. If there are significant group differences in the baseline parameter, an ANCOVA cannot be computed for that prepulse condition. You can run t-tests for group difference in the scaling parameters but be aware that these differences could be caused by non-random group differences in the baseline startle.
   c) Assuming no/few group differences in the baseline parameters, compute two ANCOVAs for each prepulse condition: one for startle-scaling as a function of group and baseline saturation, and one for sound-scaling as a function of group and baseline threshold. Include a group by baseline interaction terms in all ANCOVAs.
   d) If the baseline by group interaction terms are significant in any of the ANCOVAs, we cannot use those prepulse conditions because they break the homogeneity of slopes assumption.

e) Assuming no/few significant interaction terms, recompute all of the ANCOVAs without interaction terms, and look for significant main effects of group.
f) Control for multiple comparisons, where each of your prepulse conditions is a separate comparison, using a bootstrapped ratio test to determine the probability of seeing a given number of significant prepulse conditions by chance alone. Alternatively, control for multiple comparisons using Bonferroni correction or related methodology.
g) Report group differences in PPI startle-scaling or sound-scaling if it holds up to the control for multiple comparisons.

Results generalize across different background sounds, ages, and manipulations
We separated the experiments with a 70 dB background sound level from those with a 77 dB background sound level and separately analyzed all of our primary findings for the two background sound levels. All of the results were consistent with our original findings. In addition, the animals used for the 70 dB and 77 dB experiments were different ages. In the 70 dB experiments, the animals were 3 – 7 months old at the time of experimentation, whereas in the 77 dB experiments the animals were 9 – 15 months old. Thus, this control also shows that our results generalize across these relative age groups.

In particular, for both 70 dB background (younger rats) and 77 dB background sounds (older rats) experiments:

(1) The startle response distribution is better described by a log-normal than a normal distribution within animals for all of the stimuli. For the 70 dB background sound experiments, data from only 0.4% of all of the stimuli across all animals were consistent with a Gaussian distribution, compared to 38.38% consistent with a log-normal distribution ($p > 0.05$, Shapiro-Wilks test). Likewise, for the 77 dB background condition, 10.74% of the stimuli across all of the animals were consistent with a Gaussian distribution, compared to 62.72% consistent with a log-normal distribution ($p > 0.05$, Shapiro-Wilks test).
(2) The PPI ratio metric is inversely correlated with startle sound level. For the 70 dB background experiments, 263/288 (91.3%) of prepulse conditions across rats had a negative slope of PPI ratio versus startle sound level. For the 77 dB background experiments, 193/200 (96.5%) of prepulse conditions across rats had a negative slope of PPI ratio versus startle sound level.
(3) A model with both startle-scaling and sound-scaling has lower cross-validated normalized error than a model with just startle-scaling. The 70 dB background experiments had lower cross-validated error than the model containing one parameter to describe PPI in 55/58 (94.8%) model fits, i.e. rat-experiments. The 77 dB background experiments had lower cross-validated error than the model containing one parameter to describe PPI in 63/66 (95.5%) model fits.
(4) Both startle-scaling and sound-scaling were positively correlated with prepulse sound level, as expected for a measure of prepulse inhibition. Also, for the 70 dB experiments where we varied delay, startle-scaling and sound-scaling were inversely correlated with delay time. For the 70 dB background experiments, scaling verssus prepulse level slopes were positive for 24/24 comparisons (100 ms delay) and scaling versus delay slopes were negative for 141/156 (90.4%) comparisons (prepulse of either 14 or 18 dB). For the 77 dB background experiments, scaling versus prepulse level slopes were positive for 106/116 (91.4%) comparisons (100 ms delay).

(5) Startle-scaling was inversely correlated with baseline saturation, and sound-scaling was inversely correlated with baseline threshold. For the 70 dB background experiments, the mean Person's r for sound-scaling versus baseline threshold was -0.31 ± 0.15, and the r^2 values ranged from 0 to 0.83; the mean Pearson's r for startle-scaling versus baseline saturation was -0.47 ± 0.08, and the r^2 values ranged from 0.02 to 0.75. For the 77 dB background experiments, the mean Person's r for sound-scaling versus baseline threshold was -0.49 ± 0.08, and the r^2 values ranged from 0.01 to 0.57; the mean Pearson's r for startle-scaling versus baseline saturation was -0.60 ± 0.04, and the r^2 values ranged from 0.20 to 0.57.
(6) For the *Fmr1* KO versus WT male comparison, the 70 dB experiments were those that primarily varied the prepulse level and the 77 dB background experiments were those that primarily varied the delay time. Furthermore, as we described in response to the previous comment, we never combine data from prepulse-varying and delay-varying experiments because we do not know the correspondence between manipulations of these variables. As such, the LDA and ANCOVA analyses are already split by background level, and since none of these comparisons were significant, we can conclude that we were not able to detect group differences to either of the background sounds.
(7) All of the experiments comparing WT male and WT female rats used a 70 dB background sound, so these comparisons cannot generalize across background sound.

Animals did not startle prior to the startle sound
First, we computed the startle response in the window after the prepulse sound onset but before the startle sound onset. For 457/462 (98.9%) rat-conditions, the average startle response to the prepulse sound remained below the rat's 5% startle threshold. Thus, the vast majority of rats, in the vast majority of conditions, did not startle to any of the prepulse conditions.

Next, we analyzed the startle response for all of the animals in the 20 ms window prior to the startle sound onset, i.e. the end of the delay interval. In this period, we found that the startle response was below threshold for all 462/462 rat-conditions. Thus, even in the rare cases where there were startles during the prepulse time window, the startle responses had all returned to below 5% startle threshold before the onset of the startle sound for all rats and all prepulse conditions.

Finally, we analyzed whether the model parameters were any different for the 9/462 rat-conditions that exceeded threshold during the time of the prepulse sound. Of these 9 rat-conditions, there were 8 unique rats since one rat exceeded threshold in two conditions. For these 8 rats, we compared the baseline model parameters (saturation, slope, and midpoint) with the parameters for the other rats in comparable experiments that didn't startle to a prepulse. The absolute Z-scores were less than 2.0 for all of the 8 rats' baseline parameters, and for the slope and midpoint parameters the absolute Z-scores were always less than 1.0.

We also looked at the PPI scaling parameters. One of the 9 rat-conditions exceeding 5% threshold was to the 0 dB prepulse condition, which by definition has no scaling in our model. For each of the 8 rat-conditions with a nonzero prepulse sound, we compared the startle-scaling and sound-scaling parameters with all of the other rats at that prepulse condition. For sound-scaling 6/8 rat-conditions had an absolute Z-score below 2.0, while for startle-scaling all 8/8 the absolute Z-score was below 2.0 for all rat-conditions.

Dynamics between first and second halves of experiment

We fit the model separately to the animals' startle data from the first and last halves of an experiment (spanning a total of 12 session across several days) and then looked for changes in the model parameters for individual animals' baseline startle curves.

We detected small but significant changes in the baseline model parameters and startle threshold from the first to last halves of the experiments. Within animals, the startle saturation decreased by a mean of 6.10 ± 0.77% ($p < 10^{-12}$, t-test), which is 26.4 ± 3.2% of the between-animals IQR for this parameter. The startle threshold increased by a mean of 5.57 ± 0.86% ($p < 10^{-8}$, t-test), which is a mean of 20.2 ± 3.1% of the between-animals IQR. The slope increased by a mean of 7.57 ± 2.71% ($p < 0.03$, t-test), which is a mean of 21.8 ± 9.6% of the between-animals IQR. The midpoint increased by a mean of 2.47 ± 0.40% ($p < 10^{-8}$, t-test), which is a mean of 12.6 ± 2.0% of the between-animals IQR.

We also looked for changes in the PPI startle-scaling and sound-scaling parameters between the first and second halves of the experiments. We detected increases in startle-scaling in 6/13 prepulse conditions ($p < 0.05$, t-test), which is more conditions that we expect by chance ($p < 10^{-4}$, bootstrap test for multiple comparisons), and 4/13 hold up to Bonferroni correction for multiple comparisons ($p < 0.004$). Furthermore, the biggest changes were in the stronger prepulse conditions (louder prepulse, shorter delay). The mean startle-scaling changes across animals within the 6 conditions ranged from 2.7% - 17.9%. However, the changes for individual animals were relatively small compared to differences between animals. The mean ratio of within-animal change in startle-scaling to the across-animal standard deviation was greater than 1 in only 1/13 conditions, and in no conditions was it greater than 2.

We also found a small decrease in sound-scaling in 2/13 conditions and a small increase in sound-scaling in 1 condition, which is more conditions that we expect by chance ($p < 0.03$, bootstrap test). However, none of the conditions held up to Bonferroni correction ($p > 0.004$), the changes were not in a consistent direction, and the overall magnitude of the changes were smaller than for startle-scaling.

No evidence for hearing loss due to loudest startle sounds or older animals

As discussed above, our results hold true even if we limit ourselves to analyzing the experiments with a 70 dB background sound (and hence lower absolute sound levels), which were also the experiments with only younger animals. Thus, any potential hearing loss due to the louder sounds or older animals does not affect our main conclusions. Nevertheless, we did observe small but significant changes in the baseline parameters and startle threshold between the first and second halves of the experiments, and these changes are generally in the directions we might expect from hearing loss.

If these changes were due to hearing loss caused by the loudest sound levels or the age of the oldest animals, then we would expect to see larger magnitude changes in experiments with louder absolute sounds and older animals, compared to experiments with weaker absolute sounds and younger animals. To test this, we separately computed the changes in baseline model parameters between the first and second halves for experiments with 70 dB background sound and for experiments with 77 dB background sound. The experiments with 70 dB background sound always had lower maximal absolute sound level because the maximal relative sound level was 60 dB above background in both experiment types, and the 70 dB background experiments always had younger animals (see Supplementary Table 1).

We detected no differences in any of the baseline parameters nor in the startle threshold between the 70 dB experiments and the 77 dB experiments ($p > 0.08$, t-test), and the trend was

actually toward greater changes in the 70 dB experiments (data not shown). These results indicate that the loudest startle sounds did not in and of themselves result in significant hearing loss, even among the older animals. It is still possible that the changes observed in both experiment types could have been caused by hearing loss not attributable to the loudest sounds alone, but it is not obvious how this would occur. We therefore suggest that these changes are likely due to habituation or other dynamics of the startle response not analyzed here.

Supplementary Table 1. Experiments, cohorts, and conditions

Cohort and experiment	Rats	Ages	Primary manipulation	Prepulse conditions	Startle sound levels	Repeats per stimulus[2]	Background sound level
Fmr1 cohort 1, experiment 1	10 *Fmr1* KO & 8 WT male rats	11-12 months	Prepulse sound level	4 & 8 dB above background (at 100 ms)	0, 20, 30, 40, 45, 50, & 60 dB above background	30	77 dB
Fmr1 cohort 1, experiment 2	9 *Fmr1* KO & 7 WT male rats[1]	14-15 months	Prepulse sound level	2, 6, 10, 14, & 18 dB above background (at 100 ms)	0, 20, 25, 30, 35, 40, 50, & 60 dB above background	60	77 dB
Fmr1 cohort 2, experiment 1	9 *Fmr1* KO & 9 WT male rats	4-5 months	Delay time	50, 75, 100, & 200 ms (at 14 dB)	0, 25, 30, 35, 40, 45, 50, 55, & 60 dB above background	84	70 dB
WT male-female cohort 1, experiment 1	6 WT male & 6 WT female rats	4-5 months	Prepulse sound level	2, 6, 10, 14, & 18 dB above background (at 100 ms)	0, 20, 25, 30, 35, 40, 50, & 60 dB above background	60	70 dB
WT male-female cohort 1, experiment 2	6 WT male & 6 WT female rats	6-7 months	Delay time (at 2 prepulse sounds)	50 & 200 ms (at 14 dB); 50, 100, & 200 ms (at 18 dB)	0, 20, 25, 30, 35, 40, 50, & 60 dB above background	60	70 dB
WT male-female cohort 2, experiment 1	6 WT male & 6 WT female rats	3-4 months	Delay time (at 2 prepulse sounds)	50 & 75 ms (at 14 dB); 50, 75, & 100 ms (at 18 dB)	0, 20, 25, 30, 35, 40, 50, & 60 dB above background	60	70 dB
WT male-female cohort 2, experiment 2	6 WT male & 6 WT female rats	5-6 months	Delay time	50, 75, & 100 ms (at 14 dB)	0, 25, 30, 35, 40, 45, 50, 55, & 60 dB above background	80	70 dB

All-WT male cohort 1, experiment 1	12 WT male rats	9-11 months	Prepulse sound level	4 & 8 dB above background (at 100 ms)	0, 20, 30, 40, 45, 50, & 60 dB above background	30	77 dB
All-WT male cohort 1, experiment 2	12 WT male rats	13-15 months	Prepulse sound level	0, 2, 6, 10, 14, & 18 dB above background (at 100 ms)	0, 20, 25, 30, 35, 40, 50, & 60 dB above background	60	77 dB

1. One *Fmr1* KO rat was euthanized after developing a tumor, and one WT male rat was not included due to experimental error.
2. Stimulus is defined as a unique combination of prepulse sound level and startle sound level (e.g. 14 dB prepulse, 100 ms delay, 35 dB startle sound)

Supplementary Figure Legends:

Supplemental Figure 1 Characterization of the *Fmr1* KO rat. (a) Two mutant models were generated (Fmr1-m2 and Fmr1-m4) with frame-shift indels in exon 7. CRISPR-SpCas9 target site is underlined with protospacer adjacent motif (PAM) in bold. (b) FMR1 expression is absent in knockout male whole brain extracts. (c) Body weight trended larger, and testes/body weight ratio was greater in m2 and m4 knockout males compared to wildtype littermates at 30 days of age. N = 3 – 5 per group, p-value determined by Student's T-test.

Supplemental Figure 2 Normalizing movement data for apparatus gain. (a) Raw accelerometer data for all trials (colors) in a single session for one rat. Inset shows accelerometer data in the first 100 ms of the trials. (b) Histogram of accelerometer readings in the first 100 ms across all trials in a single session for one rat (same rat as Fig. S2a). Solid curve shows the Gaussian fit to the histogram, and the legend shows the mean and standard deviation of this Gaussian. (c) Normalized movement of a single trial for one rat (same rat as Fig. S2a&b). Dashed vertical lines indicate the startle sound onset (left) and 100 ms after the startle sound onset (right). Arrows indicate startle sound onset and the maximum normalized movement in the 100 ms window.

Supplemental Figure 3 Estimation precision of model parameters and their correlations. (a) Distribution of RMS errors for the model for each animal, compared to the distribution of errors that occur from swapping the model parameters for each rat with the parameter sets for all of the other rats in the same experiment (left), and the within-rat changes in error that occur from swapping parameter sets from other animals (right). Note that all values of the difference are greater than zero, indicating that the actual parameter set is always the better fit compared to all other animals' parameter sets within an experiment. (b) Scatter plot of sound-scaling versus threshold (left) and startle-scaling versus saturation (right) for the 14 dB, 100 ms condition from experiments that varied the prepulse level. Vertical and horizontal error bars for each point indicate 90% confidence intervals. The correlations between the x- and y-axis measures is evident even when taking into account the confidence intervals (c) Range of correlation values that occur due to resampling the parameters from within their confidence intervals by refitting the model 10,000 times to jittered data and recomputing the correlations. For the correlations observed between sound-scaling and threshold (blue), 14/15 have $p < 0.05$ of including the

value $r = 0$. For the correlations observed between startle-scaling and saturation (red), 10/15 have $p < 0.05$ of including the value $r = 0$. These data indicate that the majority of observed correlations are robust to noise in the data. (d) Correlation values between startle-scaling and baseline saturation (left) and between sound-scaling and baseline threshold (right) for prepulse-varying experiments (top) and delay-varying experiments (bottom). Points are the observed Pearson's r values across animals and within conditions (same data that makes up histograms in Fig. 4d). Boxes are the median and interquartile range (IQR) of the r values within animals and within conditions after jittering the parameters by the noise in the data (i.e. representing what could be expected for the correlations just due to compensatory effects in the parameters that would be exposed solely due to noise in the data). For panels c & d, whiskers extend to the last datum within 1.5 IQR beyond the first and third quartiles; points outside of this range are represented as outliers. Dotted horizontal line represents an r value of 0.

Supplemental Figure 4 First principal component captures significant variance in the model parameters. (a) Eigenvalues of the four principal components (PCs) of the reduced set of model parameters (startle-scaling, sound-scaling, saturation, and threshold) across all WT male rats for the prepulse condition with 14 dB prepulse and 200 ms delay. Grey curve shows mean and standard deviation of the eigenvalues across random permutations of the parameters. (b) P-values of the first PC eigenvalue from a permutation test on the parameter values, across all prepulse conditions. Dashed horizontal line indicates p = 0.05.

Supplemental Figure 5 PPI is more sensitive to differences in baseline startle with louder prepulse sounds and shorter delays. (a) Slope of the sound-scaling versus baseline threshold regression slopes as a function of prepulse sound level at a constant 100 ms delay (top); as a function of delay at a constant prepulse level of 14 dB above baseline (middle); and as a function of delay at a constant prepulse level of 18 dB above baseline (bottom). (b) Slope of the startle-scaling versus baseline saturation regression slopes as a function of prepulse level at a constant 100 ms delay (top); as a function of delay at a constant prepulse level of 14 dB above baseline (middle); and as a function of delay at a constant prepulse level of 18 dB above baseline (bottom).

Supplemental Figure 6 No difference in startle-scaling or sound-scaling between *Fmr1* KO and WT male rats. (a) Linear regressions of sound-scaling versus baseline threshold for *Fmr1* KO male (red) and WT male (blue) rats from experiments that varied the prepulse sound level with a constant 100 ms delay. Subplots show increasing prepulse sound level from left to right. (b) Linear regressions of sound-scaling versus baseline threshold for *Fmr1* KO male rats and WT male rats from experiments that varied delay with a constant 14 dB prepulse. Subplots show decreasing delay from left to right. (c) Linear regressions of startle-scaling versus baseline saturation for *Fmr1* KO male rats and WT male rats from experiments that varied the prepulse sound level with a constant 100 ms delay. Subplots show increasing prepulse sound level from left to right. The black asterisks indicate the prepulse condition where *Fmr1* KO rats had a lower startle-scaling than WT rats (p < 0.05, ANCOVA). *Fmr1* KO rats had lower startle-scaling than WT rats in 1/11 prepulse conditions, which is not significant after multiple comparisons (p > 0.05, bootstrap ratio test). (d) Linear regressions of startle-scaling versus baseline saturation for *Fmr1* KO male rats and WT male rats from experiments that varied delay with a constant 14 dB prepulse. Subplots show decreasing delay from left to right. For a, b, c, & d there were no group

differences in baseline parameters ($p > 0.05$, t-test), no baseline by group interactions ($p > 0.05$, ANCOVA), and no group differences startle-scaling or sound-scaling at any prepulse condition ($p > 0.05$, ANCOVA).

Supplemental Figure 1

a

```
ATCTGATGGGTCTAGCTATTGGTACTCATGGTGCTAATATTCAGCAAGCT - WT Fmr1
ATCTGATGGGTCTAGCTATTGGTAGCATCATGGTGCTAATATTCAGCAAGCT - Fmr1-m2
ATCTGATGGGTCTAGCTATTGGTA--CATGGTGCTAATATTCAGCAAGCT - Fmr1-m4
```

b

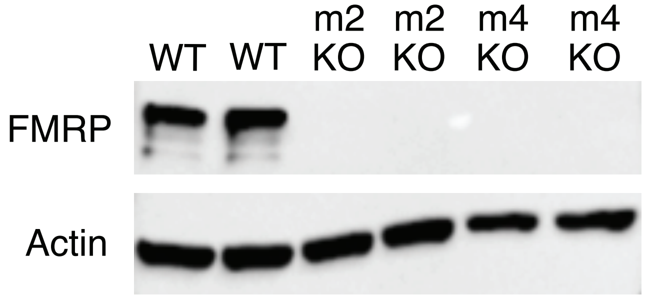

c

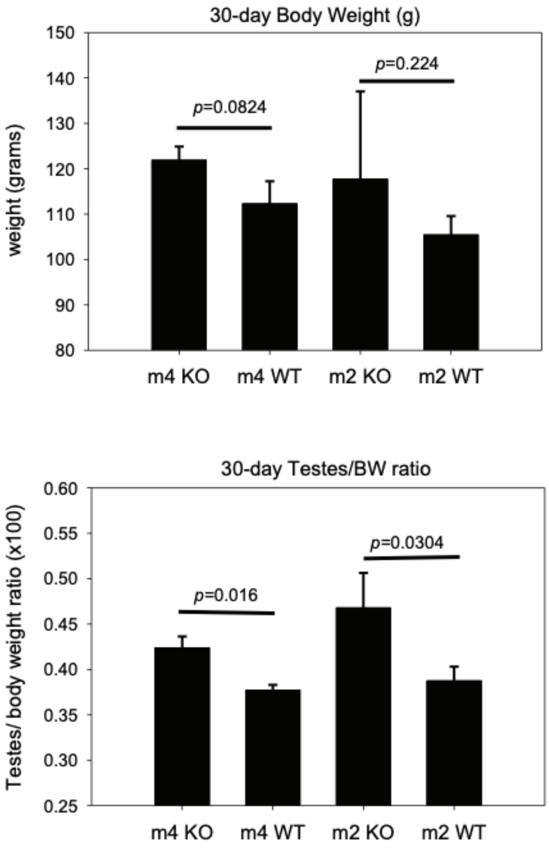

Supplemental Figure 2

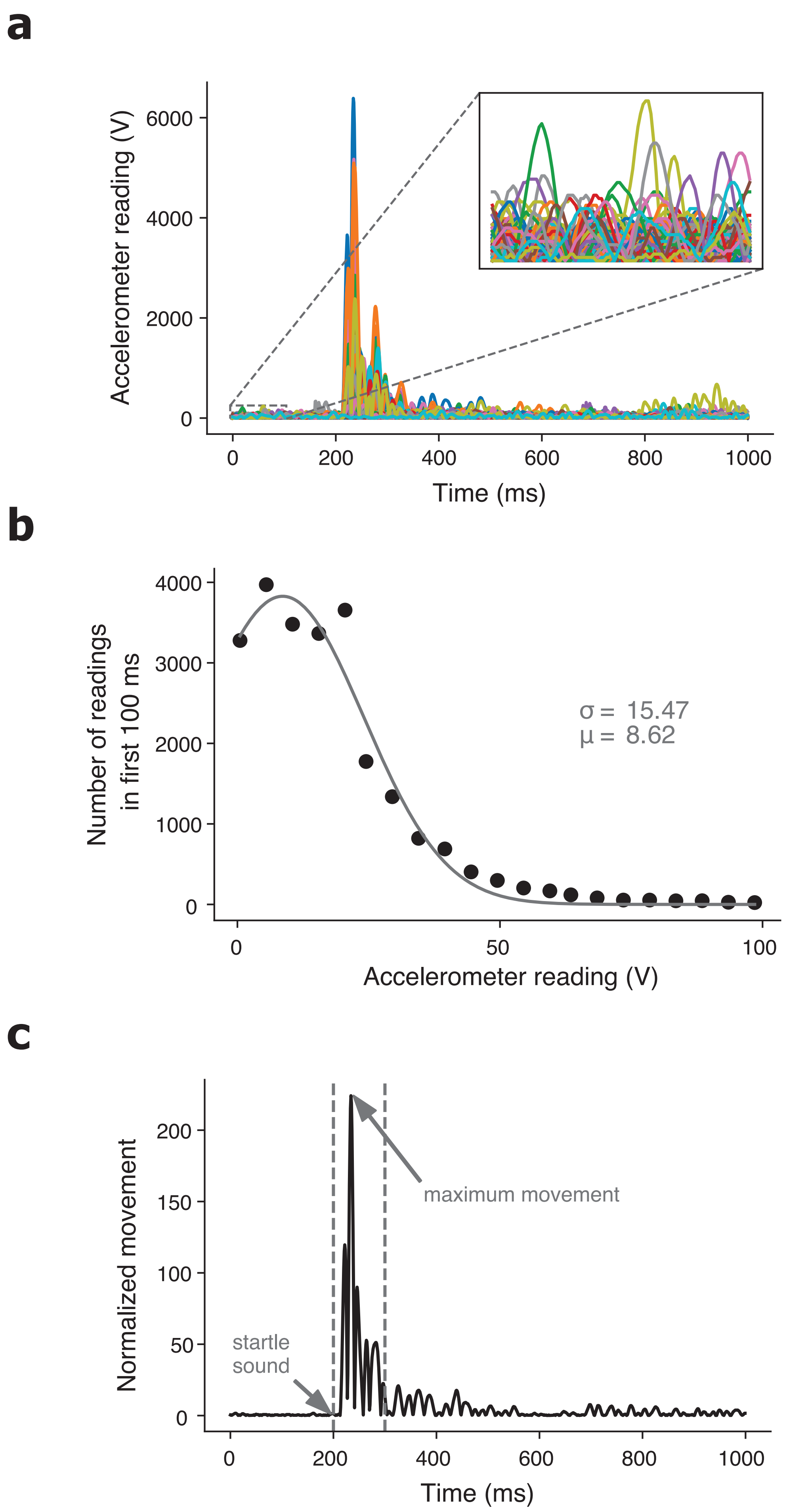

Supplemental Figure 3

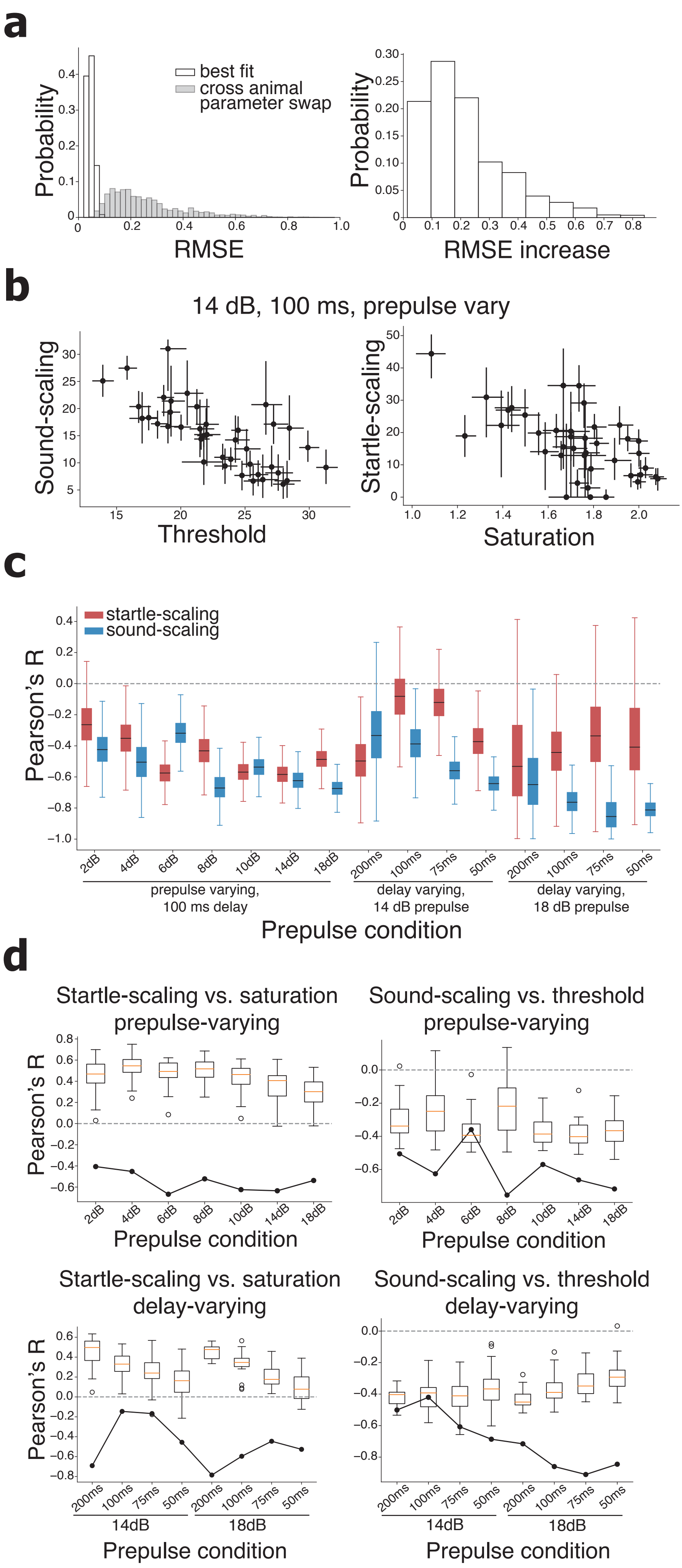

Supplemental Figure 4

a

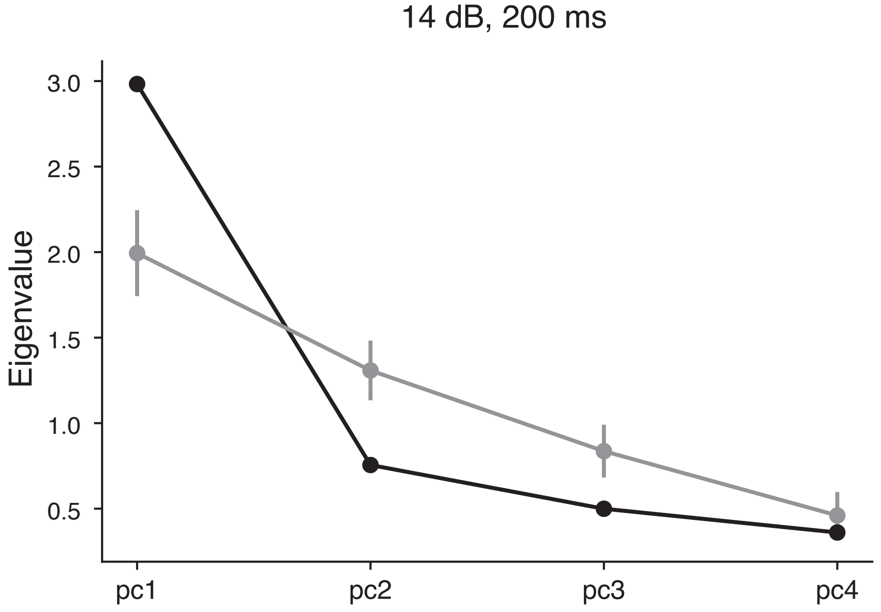

b

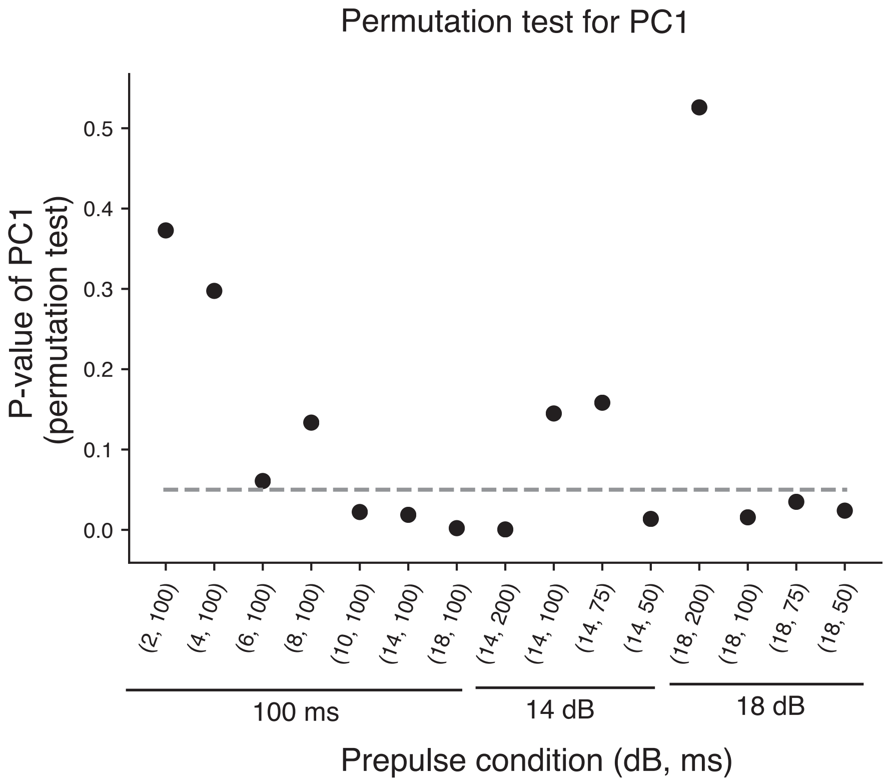

Supplemental Figure 5

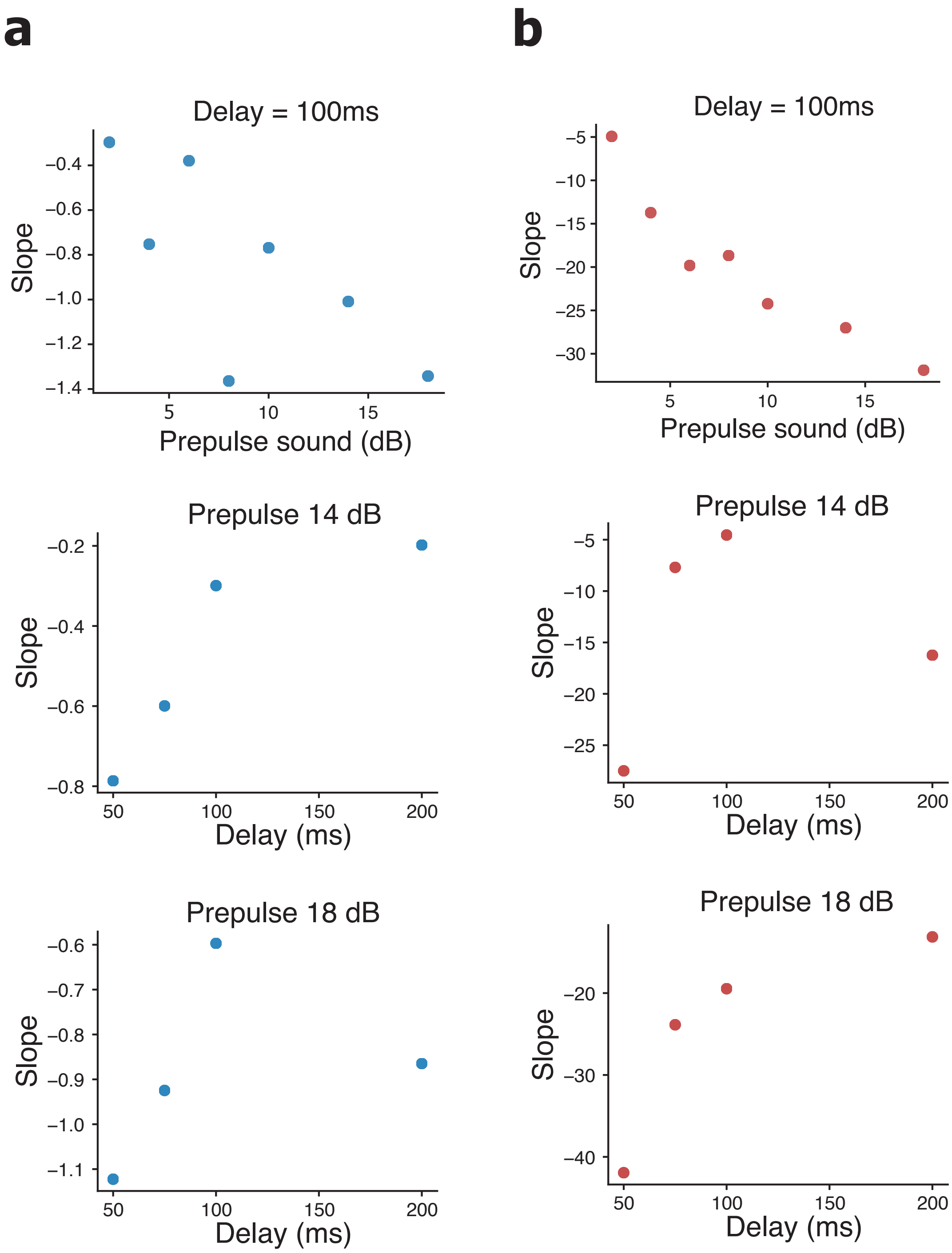

Supplemental Figure 6

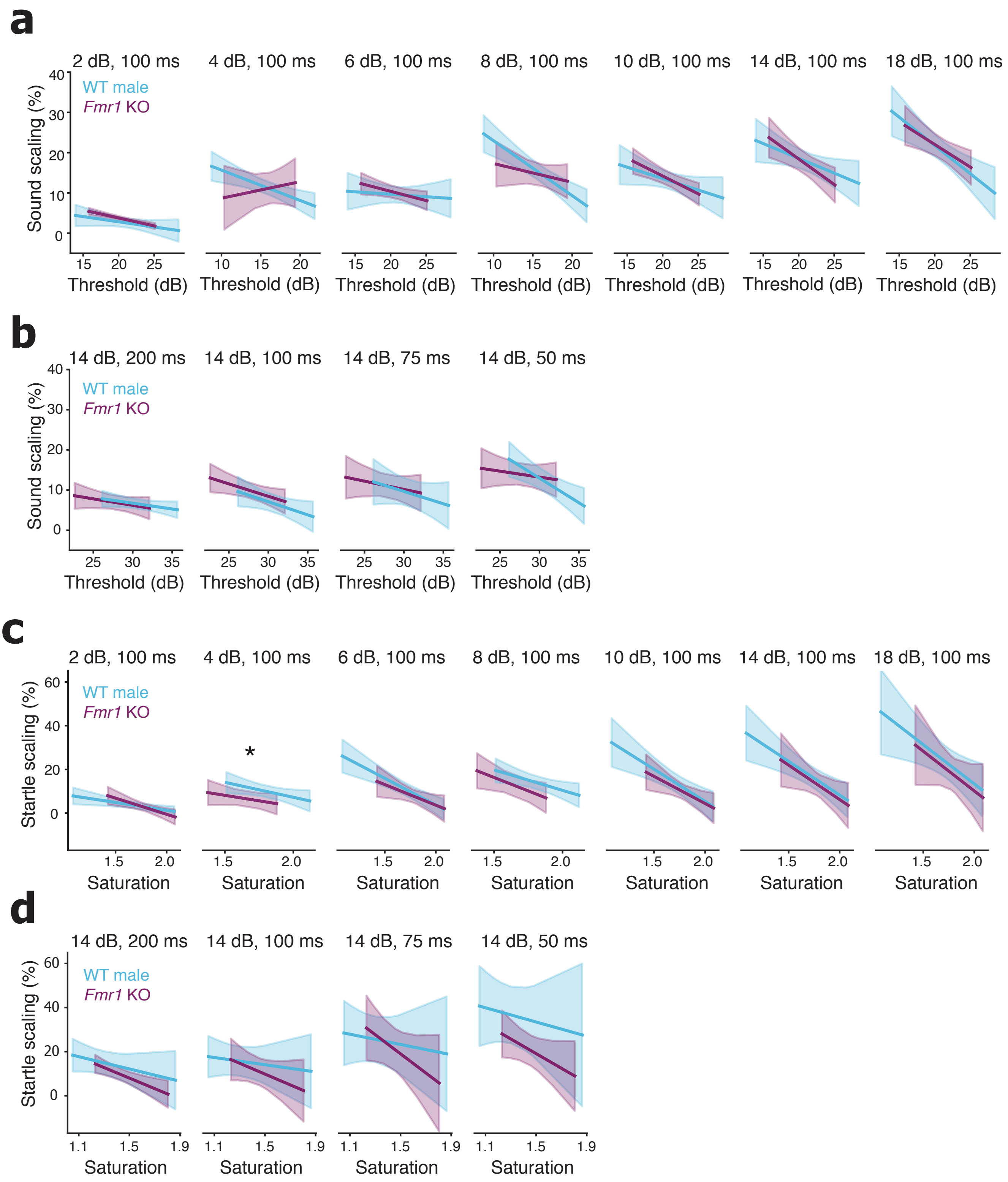

Supplement: Supplementary file 1 — Supplemental Material [file 41380_2020_703_MOESM1_ESM.pdf]
